# An MRI assessment of mechanisms underlying lesion growth and shrinkage in multiple sclerosis

**DOI:** 10.1002/acn3.52308

**Published:** 2025-01-27

**Authors:** Ermelinda De Meo, Ferran Prados Carrasco, J. William L. Brown, Alasdair J. Coles, Nick G. Cunniffe, Amy E. Jolly, Baris Kanber, Rebecca Samson, Frederik Barkhof, Declan Chard

**Affiliations:** ^1^ NMR Research Unit, Queen Square Multiple Sclerosis Centre, UCL Queen Square Institute of Neurology University College London London UK; ^2^ Universitat Oberta de Catalunya Barcelona Spain; ^3^ Department of Medical Physics and Biomedical Engineering, Centre for Medical Image Computing (CMIC) University College London London UK; ^4^ Department of Clinical Neurosciences University of Cambridge Cambridge UK; ^5^ National Institute for Health Research (NIHR) University College London Hospitals (UCLH) Biomedical Research Centre London UK; ^6^ Radiology and Nuclear Medicine Amsterdam UMC Amsterdam the Netherlands

## Abstract

**Objective:**

To assess the pathological mechanisms contributing to white matter (WM) lesion expansion or contraction and remyelination in multiple sclerosis (MS).

**Methods:**

We assessed 1,613 lesions in 49 people with relapsing–remitting MS in the CCMR‐One bexarotene trial (EudraCT 2014‐003145‐99). We measured lesion orientation relative to WM tracts, surface‐in gradients and veins. Jacobian deformation was used to assess lesion expansion over 6 months, while magnetization transfer ratio (MTR) imaging was used to assess remyelination.

**Results:**

At baseline, 33% of lesions were aligned with veins, 2% along WM tracts, 0% with surface‐in gradients, and 4% orthogonal to veins. No significant differences were observed in lesion shape, while lesions aligned with surface‐in gradients and with veins had lower volume compared to all remaining orientations. At follow‐up, 13% of lesions expanded and 7% contracted. The directions for both expansion and contraction were 18% and 8%, respectively, along WM tracts, 20% and 15% parallel to veins, 22% and 23% orthogonal to veins and 0% and 1% along surface‐in gradients. Bexarotene had no effect on lesion expansion or contraction, but MTR significantly increased in lesions aligned with surface‐in gradients and veins.

**Interpretation:**

Lesion expansion and shrinkage are affected by venous and WM tract factors, but these do not influence bexarotene's capacity to promote remyelination. This, instead, appears to be affected by surface‐in factors. To limit lesion expansion and maximize tissue repair, multiple processes may need to be targeted.

## Graphical abstract

By applying the tensor model, we analysed lesion orientation and the directionality of lesion expansion/contraction in multiple sclerosis. Each lesion is summarized as an ellipsoid, and the tensor model is applied to calculate lesion anisotropy. From the top to the bottom white matter atlas, surface‐in gradient segmentation and venous atlas used in the present study are represented together with examples of lesions aligned with each orientation assessed.

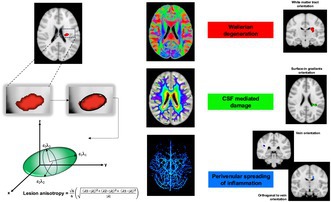

## Introduction

Focal inflammatory demyelinating white matter (WM) lesions are the most recognized feature of multiple sclerosis (MS). Their formation is venocentric and characterized by acute inflammation with the breakdown of the blood–brain barrier, demyelination and acute axonal transection, followed by a resolution of inflammation and variable degrees of remyelination over weeks to months. Mirroring this, lesions expand in the acute phase and then contract as inflammation resolves and repair occurs.^1^ However, recently, it has been recognized that a proportion (up to 53% in relapsing–remitting (RR)MS and up to 62% in progressive (P)MS)^2^, ^3^ of WM lesions may continue to show signs of chronic inflammatory activity over years.^4^, ^5^

Paramagnetic rim lesions (PRLs) identified using susceptibility‐based imaging (SWI) by the presence of paramagnetic rims^6^ have been proposed as an in vivo biomarker for chronic inflammatory activity within these lesions. Histopathologically, these lesions are characterized by a hypocellular core and activated iron‐enriched macrophages–microglia at the lesion border that can lead to expansion, further myelin damage, axonal loss and gliosis.^1^, ^5^, ^7^ Another in vivo biomarker for chronically active lesions is their expansion over time, called slowly evolving lesions (SELs).^8^ However, there is only partial overlap between PRLs and SELs.^9^, ^10^

MS lesions are not spherical, and their shape can be influenced by their location. For example, in contrast to periventricular Dawson's fingers, deep WM and juxtacortical lesions are typically less elongated. However, other factors could influence subsequent lesion expansion, including WM tract‐mediated processes (e.g. axonal degeneration),^11^ surface‐in pathological gradients (possibly linked with diffusible agents from the CSF)^12^, ^13^ and perivenular collagenosis hampering tissue repair,^14^ but it is not known how relevant these are to chronic lesion activity (and so expansion) or in limiting remyelination (and potentially lesion contraction). Given the known association between SELs and clinical disability,^15^ understanding the mechanisms underlying lesion dynamic changes could be relevant as we develop treatments to prevent or slow chronic lesion expansion and promote repair. It may also impact significantly the way in which we measure treatment responses in early phase trials, as directional effects on lesion expansion or contraction may be overlooked in directionless volume measures.

Based on the potential mechanisms noted earlier, in the present study, we assessed WM lesion orientation with respect to WM tracts, CSF surface‐in gradients and veins. We explored the differences in lesion shape, volume and microstructure based on this, and whether WM lesion expansion and contraction showed a directional preference. We then assessed whether lesion orientation had any detectable influence on lesion expansion, contraction or remyelination [as measured using magnetization transfer ratio (MTR)] following bexarotene therapy (which has already shown a remyelinating effect on MRI‐derived lesion measures).^16^

## Methods

### Participants

We retrospectively analysed data from the Cambridge Centre for Myelin Repair Trial Number One (CCMR‐One, ISRCTN14265371). CCMR‐One was a double‐blind phase 2a trial in people with relapsing–remitting multiple sclerosis from two UK centres, aged 18–50 years who had been stable on dimethyl fumarate for at least 6 months. Full inclusion criteria, baseline demographics and methodology can be found in the original trial manuscript.^16^ Briefly, participants were randomized to receive bexarotene (300 mg/m^2^) or placebo tablets for 6 months and underwent MRI at baseline and 6 months. The trial was approved by the London Westminster National Research Ethics Service Committee (15/LO/0108) and all participants gave written informed consent at enrolment.

### MRI acquisitions

A Siemens 3T Prismafit scanner (Siemens, Erlangen, Germany) was used at each site with 20‐channel head–neck coils. The following sequences were obtained: 3D magnetization transfer imaging, 3DT1‐weighted, proton density/T2‐weighted, fluid‐attenuated inversion recovery and post‐gadolinium T1‐weighted spin echo. Detailed acquisition parameters are available in Supplementary Material.

### MRI analyses

#### MTR maps reconstruction

The MTR maps [in percentage units (pu)] at baseline and 6‐month follow‐up were calculated directly as follows: [((MToff − MTon)/MToff) × 100].

#### WM lesion segmentation

The lesion and tissue segmentation processing is detailed in the original trial manuscript^16^ and in Supplementary Material.

#### WM lesion orientation

We determined lesion orientation relative to atlases of the feature of interest (see below) in the MNI 152 template space. To avoid changes in the shape of the lesions, we rigidly registered lesion‐filled 3D T1‐weighted brain images (so that periventricular lesions were filled and the ventricular boundary clear) to the MNI 152 template and then applied the same transformation to the lesion mask, which was then used as input for the regionprops3 toolbox (Matlab 2023a).^17^ This toolbox, summarized each lesion as an ellipsoid, allowed us to identify the lesion centre of mass, the eigenvalues (λ_1_, λ_2_, λ_3_) (corresponding to ellipsoid major axis lengths) and the related eigenvectors (ε_1_, ε_2_, ε_3_) (corresponding to ellipsoid major axis orientation). The main lesion orientation was identified by the eigenvector ε_1_, corresponding to the lesion major axis (first eigenvalue λ_1_).

By applying the diffusion tensor scheme, we calculated lesion anisotropy as follows:

$$\text{Lesion anisotropy}=\frac{\surd 6}{6}\left(\sqrt{\frac{{\left(\lambda 1-|\lambda \right)}^{2}+{\left(\lambda 2-|\lambda \right)}^{2}+{\left(\lambda 1-|\lambda \right)}^{2}}{\left|\lambda \right|.}}\right).$$

#### Surface‐in gradients

Starting from the lateral ventricles of the MNI 152 template, we calculated the surface‐in gradient direction within each voxel as the direction orthogonal to the ventricular surface. For each voxel on the ventricular surface, we first determined the tangent plane. Then, we calculated the gradient vector ∇ƒ, which is perpendicular to the tangent plane of the surface at that voxel. This provided us with the eigenvector representing the surface‐in gradient direction along the three Cartesian axes for each voxel. By averaging the eigenvector components along each of the three axes across all the voxels within a lesion, we derived the mean surface‐in gradient direction within each lesion.

#### WM tract direction

To identify mean WM tract direction within each lesion, we intersected lesion masks in MNI 152 space with the primary eigenvector (ε_1_) map from the Human Connectome Project (HCP)1065 standard‐space diffusion tensor imaging (DTI) template, which is associated with the largest eigenvalue (λ_1_) of the diffusion tensor, and typically aligns with the dominant orientation of white matter fibres.^18^, ^19^ We determined WM tract direction within each voxel and then calculated the mean WM tract direction superimposed onto the voxels within each lesion.

#### Vein direction

To identify the vein direction within each lesion, we intersected each lesion mask with a vein atlas obtained from the susceptibility weighted images (SWI) of 42 healthy people.^20^ We identified the segmented veins within each lesion, binarized them and assessed their direction again using the regionprops3 toolbox (Matlab 2023a).^17^ By adopting this approach, we obtained the direction of veins within each lesion expressed as an eigenvector along the three Cartesian axes. In addition, to test the hypothesis of perivenular collagenosis as the mechanism underlying lesion expansion, we also assessed a fourth direction orthogonal to the principal axis of veins. The Graphical Abstract summarizes the study pipeline, providing examples of lesions aligned with each orientation.

#### Lesion directionality definition

By applying the formula for the angle between two vectors, we identified the angle between the lesion major axis and surface‐in gradient, WM tract, venous and orthogonal to venous directions within each lesion as follows:

$$\cos\left(\theta \right)=\frac{a∙b}{\left|a\right|\left|b\right|}.$$

A lesion whose major axis was <45° of a feature's direction (e.g. a WM tract) was attributed to that direction. Lesions could be classified as being orientated with more than one feature simultaneously, where those features were also aligned, for example, surface‐in gradients and veins around lateral ventricles. Considering this overlap between the different directions, we identified 11 orientations: (1) surface‐in gradients (G), (2) surface‐in gradients and veins (GV), (3) WM tracts (W), (4) WM tracts and veins (WV), (5) veins (V), (6) surface‐in gradients and WM tracts (GW), (7) surface‐in gradients, WM tracts and veins (GWV), (8) orthogonal to veins (V90), (9) surface‐in gradients and orthogonal to veins (GV90), (10) WM tracts and orthogonal to veins (W90) and (11) surface‐in gradients, WM tracts and orthogonal to veins (GW90). Lesions whose major axis did not form an angle <45° with any of the directions assessed were categorized as lesions without a specific orientation. We summarize the main results based on mutually exclusive orientations (i.e. lesions only orientated with one feature or interest) but give the results for shared orientations in Supplementary Materials.

#### WM lesion expansion

To assess lesion expansion, we computed the Jacobian determinant of the non‐linear deformation field between baseline and 6‐month scan. By adopting this approach, we were able to detect and quantify subtle changes on a per‐voxel basis. Although this analysis has been mostly used in measuring growth and shrinkage of brain structures and regions of interests,^21^, ^22^, ^23^ it has also been applied to identify SEL candidates.^8^ The Jacobian analysis pipeline used is based on that of Nakamura et al.^23^ To reduce the potential for random measurement noise in lesions that were actually volumetrically stable being spuriously classified as expanding or contracting, lesions were classified as expanding or contracting if the average Jacobian determinant in a given lesion was, respectively, ≥1 or ≤1 standard deviation (SD) from the mean of the Jacobian determinant of all lesions in the study cohort. This threshold is in line with those applied in previous studies to identify significant volume increases, for example,^24^ corresponding to the 8% increase/decrease of volume in 6 months.

#### WM lesion direction of expansion

From the deformation field, we obtained for each voxel the direction and the magnitude of deformation along the three Cartesian axes. Then, we identified the major axis of expansion, and we computed the angle between the major axis of expansion and the axis of surface‐in gradient, WM tract, venous and orthogonal to the venous direction. Lesions whose major axis of expansion was <45° of the axis of a feature of interest were attributed to that direction. We chose this inclusive 45° threshold because it is exactly halfway between a direction parallel and one perpendicular to a specific direction, allowing us to classify all lesions as either being oriented with or not oriented with a given feature. A smaller angle would reduce the risk of measurement noise leading to lesions close to the 45° cut‐off being misclassified but would also reduce the number of lesions being assessed, and the threshold itself will not materially alter conclusions about the relative proportion of lesions in any given orientation. To determine if the 45° materially influenced the results, we also assessed with a 30° threshold.

#### Random orientation probability

Given that a lesion can assume any orientation and so by chance alone align with a given feature, considering the geometrical properties of a sphere and spherical sector, we calculated the probability for a lesion to form an angle <45° with a specific feature's axis by chance alone. As there is also potential for overlap between the four specific orientations we assessed, we also calculated voxel by voxel the probability of this happening by chance alone. Further details about the calculation of random orientation probability are provided in Supplementary Materials.

### Statistical analysis

To account for inter‐individual variability in lesion orientation, the proportion of lesions orientated along each direction was computed for each participant. Fisher's exact test was used to compare the frequency of categorical variables. Linear mixed effect models with patient random intercept were used to assess the relationship of lesion features with clinical variables and to compare lesion features between expanding and contracting lesions, and lesions grouped according to their orientation. Four binary minimization factors (age [≤40 years or >40 years], sex [male or female], the trial centre [Cambridge or Edinburgh] and EDSS score [≤4.0 or >4.0]) were used as covariates, consistently with previous studies conducted on the same cohort.^16^, ^25^ When exploring longitudinal changes in lesion MTR values, we added to the above specified covariates the baseline MTR value. Linear regression models including the above specified covariates were used to assess the associations between the patient's percentage of lesions aligning with each orientation and EDSS score and its changes over time and the patient's percentage of lesions expanding and contracting along each direction and EDSS changes over time. For all analyses, the statistically significant threshold was set at p‐value <0.05.

## Results

### Demographic and clinical features

Between January 2017 and May 2019, 52 participants were randomly assigned to receive either bexarotene (*n* = 26) or placebo (*n* = 26). Two participants who were randomly assigned to the placebo group were withdrawn before receiving the placebo, and another participant randomly assigned to bexarotene withdrew consent at month 2. The mean age at study entry was 39.2 ± 6.6 years, with 57% of participants being female. The mean disease duration was 9.7 ± 5.9 years, and the median EDSS was 2.0 (interquartile range 1.5–3.5). No significant differences were observed in demographic and clinical features between the treatment and placebo groups. No significant changes were observed in EDSS scores during the follow‐up time. More details are reported in the original trial manuscript.^16^

### Lesion analysis

From 49 participants with baseline and follow‐up imaging of sufficient quality, we analysed 1,613 T2‐hyperintense pure white matter lesions with a minimum volume of 3 mm^3^. None of these lesions were gadolinium‐enhancing at either baseline or the 6‐month follow‐up. Due to the limitation of susceptibility‐weighted imaging (SWI) in detecting small‐caliber veins, it was not possible to assess vein direction in 321 lesions using the MNI atlas, leaving 1292 pure WM lesions in the final cohort. The lesions excluded, compared with those that were not, had a smaller volume, higher MTR and lower morphological anisotropy. More details are available in Table S1.

### Baseline lesion features

The mean lesion volume per participant was 65.39 ± 28.24 mL. We observed a significant association between lesion anisotropy with lesion volume (*β* = 0.18, *p* < 0.001) and disease duration (lesion anisotropy increased with disease duration, *β* = 0.003, *p* < 0.001), whereas no associations were observed between the remaining lesion features with demographic or clinical measures.

### Baseline lesion orientation

**Figure 1:**
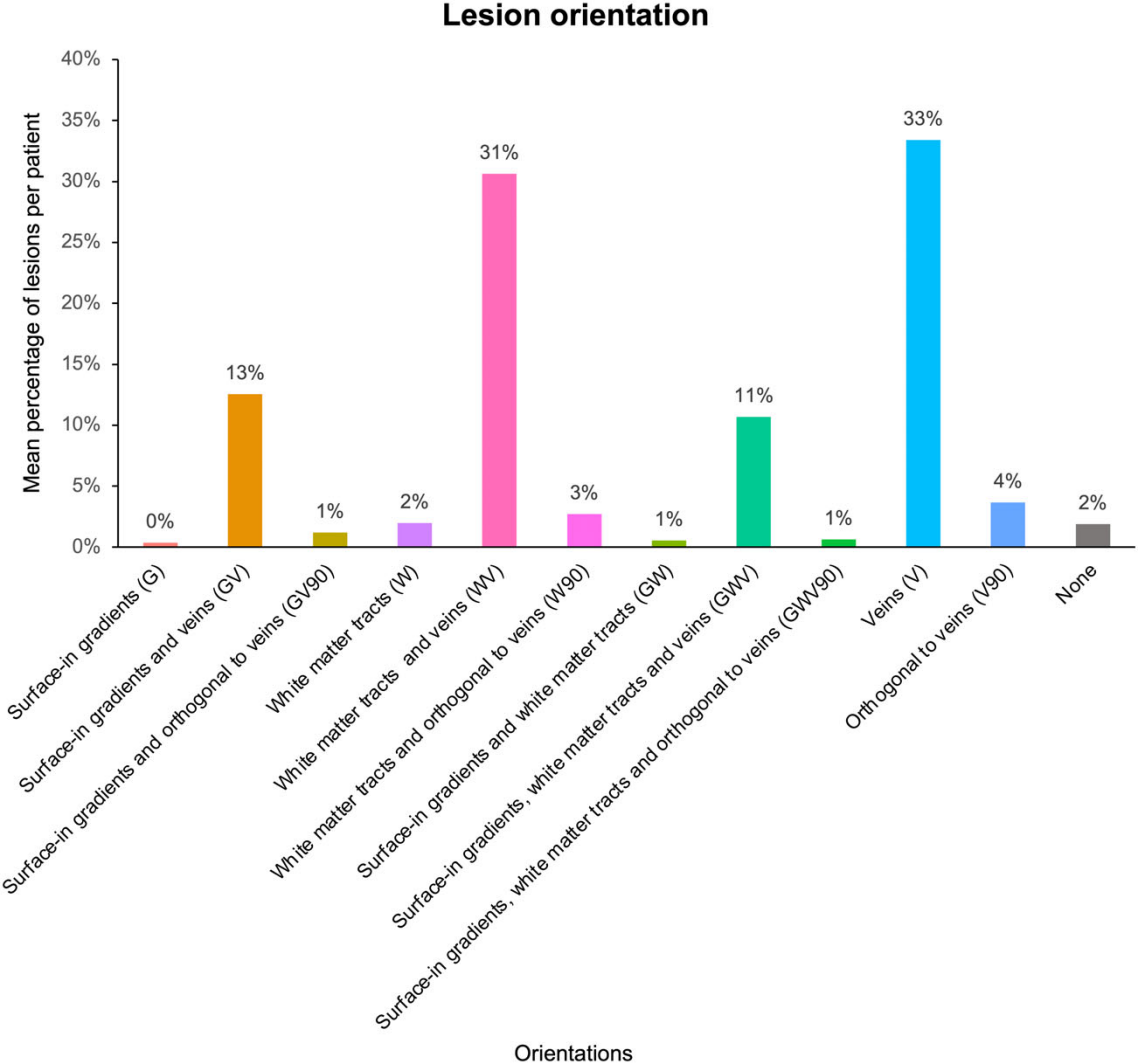
Distribution of lesions according to their orientation. The barplots summarize the mean percentage of lesions per patient for each orientation.

Among the four main orientations considered, excluding shared alignments, we observed 0% of lesions per patient orientated along surface‐in gradients (G), 2% orientated along WM tracts (W), 33% orientated along veins (V) and 4% orientated orthogonal to veins (V90). Among the shared alignments, the most frequent one was WM tracts and veins (WV) with 31% of lesions per patient aligned with this orientation, followed by surface‐in gradients and veins (GV) with 13% of lesions per patient and surface‐in gradients, WM tracts and veins (GWV) with 11% of lesions per patient. Figure 1 summarizes the mean percentage of lesions per patient for each orientation. Table 1 summarizes the mean percentage per patient of lesions orientated along the four main orientations, and the shared alignments across them, together with the probabilities for a lesion to be aligned with each feature by chance alone. Compared to the expected orientations by chance alone, when considering those exclusively aligned with a feature, lesions were orientated more frequently along veins and less frequently orthogonally to veins, parallel WM tracts and along surface‐in gradients. Please see Figure S1 and Table S2 comparing the proportions of lesions orientated with a given feature based on a 30° rather than 45° threshold. Overall, the 30° threshold increased the proportion of lesions with no dominant orientation but did not materially change the relative proportion of lesions aligned with WM tracts, surface‐in gradients or veins. No associations were found between patient's percentage of lesions aligned with each orientation and EDSS score or its changes over time.

**Table 1:** Percentages of chance alone and observed lesion orientations at baseline.

	Expected orientation probability by chance				Observed orientation probability			
	Surface‐in gradients (G) (%)	Parallel to WM tracts (W) (%)	Parallel to veins (V) (%)	Orthogonal to veins (V90) (%)	Surface‐in gradients (G)	Parallel to WM tracts (W)	Parallel to veins (V)	Orthogonal to veins (V90)
Exclusive orientation	10	10	15	15	0% (less) (<0.001)	2% (less) (<0.001)	33% (more) (<0.001)	4% (less) (<0.001)
Overall orientation	29	29	29	29	26% (less) (0.02)	46% (more) (<0.001)	87% (more) (<0.001)	8% (less) (<0.001)
Shared alignment with other directions								
Surface‐in gradient (G)	–	5	6	6	–	1% (less) (<0.001)	13% (more) (<0.001)	1% (less) (<0.001)
WM tracts (W) Surface‐in gradient and WM tracts (GW)	–	–	4	2	–	–	31% (more) (<0.001)	3% (more) (0.01)
Surface‐in gradient and WM tracts (GW)	–	–	1	1	–	–	11% (more) (<0.001)	1% (0.99)

Overall lesion orientations include all lesions aligned with a given feature even if they are also aligned with other features. Exclusive lesion orientation only includes lesions aligned with a given feature alone.

WM, white matter.

### Lesion features according to lesion orientation

Table 2 summarizes lesion features in lesions grouped according to their mutually exclusive orientations. Compared to all the remaining lesions, those orientated along surface‐in gradients (G) and those orientated along veins (V) had lower lesional volumes, whereas no significant differences were observed compared to the lesions orientated along the remaining mutually exclusive orientations. Compared to all the remaining lesions, but not compared to the lesions orientated along the remaining mutually exclusive orientations, those orientated along surface‐in gradients (G) had higher MTR. Lesions orientated along veins (V) had higher anisotropy compared to all the remaining lesions and the other mutually exclusive orientations. Table S3 and Figure S3 summarize lesion features and between‐group comparisons between all the orientations, including shared alignments.

**Table 2:** Baseline lesion features in lesions grouped by their orientation.

Adjusted difference (95%CI) *p* values					
Lesion volume (mL)	Mean (SD)	Versus all remaining groups	Versus WM tracts (W)	Versus veins (V)	Versus 90° to veins (V90)
*N* = 7 Surface‐in gradients (G)	25.75 (20.27)	−35.00 (−69–50, −0.43) 0.05	−17.29 (−77.71, 43.15) 0.98	−22.90 (−76.13, 30.23) 0.90	−15.43 (86.69, 55.82) 0.67
*N* = 24 WM tracts (W)	43.79 (39.57)	−18.30 (−39.10, 2.51) 0.08		−5.61 (−38.43, 27.09) 0.99	−1.36 (45.21, 42.49) 0.95
*N* = 446 Veins (V)	46.71 (50.91)	−18.10 (−27.10, −9.16) <0.001			−0.55 (−1.43, 0.32) 0.21
*N* = 52 90° to veins (V90)	39.65 (81.85)	23.8 (−1.57, 49.2) 0.07			

Adjusted difference (95%CI) *p* values					
Lesion MTR (pu)	Mean (SD)	Versus all remaining groups	Versus WM tracts (W)	Versus veins (V)	Versus 90° to veins (V90)
*N* = 7 Surface‐in gradients (G)	44.78 (2.76)	1.58 (0.25, 2.92) 0.02	1.10 (−1.27, 3.47) 0.86	1.36 (−0.73, 3.44) 0.50	0.47 (1.93, 2.86) 0.70
*N* = 24 WM tracts (W)	43.86 (3.24)	0.50 (−0.31, 1.31) 0.23		0.26 (−1.02, 1.55) 1.00	−0.14 (−1.62, 1.34) 0.85
*N* = 446 Veins (V)	43.74 (3.42)	0.32 (−0.03, 0.67) 0.07			−0.55 (−1.43, 0.32) 0.21
*N* = 52 90° to veins (V90)	44.15 (2.80)	0.80 (−0.05, 1.65) 0.06			

Adjusted difference (95% CI) *p* values					
Lesion anisotropy	Mean (SD)	Versus all remaining groups	Versus WM tracts (W)	Versus veins (V)	Versus 90° to veins (V90)
*N* = 7 Surface‐in gradients (G)	0.56 (0.12)	−0.10 (−0.18, −0.03) 0.005	−0.01 (−0.14, 0.11) 1.00	−0.11 (−0.22, −0.00) 0.04	−0.02 (−0.14, 0.10) 0.74
*N* = 24 WM tracts (W)	0.58 (0.20)	−0.10 (−0.14, −0.05) <0.001		−0.09 (−0.16, −0.03) <0.001	−0.01 (−0.08, 0.07) 0.81
*N* = 446 Veins (V)	0.67 (0.16)	0.01 (−0.01, 0.02) 0.56			0.13 (0.09, 0.17) <0.001
*N* = 52 90° to veins (V90)	0.55 (0.16)	−0.13 (−0.17, −0.08) <0.001			

CI, confidence interval; MTR, magnetization transfer ratio; SD, standard deviation.

### Baseline lesion features and probability of expansion or contraction

On average, over 6 months, 13% of lesions per patient were classified as expanding and 7% as contracting. Table 3 summarizes the association between baseline lesion features (including lesion volume, MTR, anisotropy and orientation) and the probability of expansion or contraction over time. Larger individual lesion volume and anisotropy, and orientation along WM tracts (W) were associated with higher probability for a lesion to remain stable than expand over time. Higher lesion MTR values and orientation along surface‐in gradients (G) were associated with higher probability for a lesion to contract than to remain stable over time. Lesions orientated along veins (V) had higher probability to expand rather than to remain stable or contract over time.

**Table 3:** Baseline lesion features predicting lesion expansion or contraction over the 6‐month follow‐up.

Predictors	Expanding vs. Stable		Expanding vs. Contracting		Contracting vs. Stable	
	Odds ratio (95% CI)	*p* values	Odds ratio (95% CI)	*p* values	Odds ratio (95% CI)	*p* values
Lesion volume	0.72 (0.60, 0.86)	<0.001	0.85 (0.64, 1.14)	0.27	0.93 (0.24, 3.63)	0.91
Lesion MTR	1.09 (0.97, 1.23)	0.15	0.94 (0.76, 1.16)	0.54	1.15 (1.03, 1.31)	0.01
Lesion anisotropy	0.36 (0.21, 0.65)	<0.001	0.19	0.007	0.92 (0.49, 1.82)	0.83
Lesion orientation						
Surface‐in gradients (G)	0.95 (0.43, 2.11)	0.89	0.68 (0.15, 3.10)	0.62	1.07 (0.10, 11.00)	0.05
Surface‐in gradients and veins (GV)	0.98 (0.72, 1.34)	0.90	0.83 (0.47, 1.47)	0.52	1.06 (0.40, 2.81)	0.90
Surface‐in gradients and 90° to veins (GV90)	1.57 (0.24, 6.18)	0.56	1.08 (0.08, 26.48)	0.83	1.00 (0.05, 5.41)	0.99
WM tracts (W)	0.42 (0.21, 0.85)	0.01	0.70 (0.13, 3.80)	0.68	0.81 (0.09, 7.70)	0.86
WM tracts and veins (WV)	0.74 (0.60, 0.925)	0.008	0.67 (0.41, 1.02)	0.06	0.98 (0.47, 2.05)	0.95
WM tracts and 90° to veins (WV90)	NA	NA	NA	NA	NA	NA
Veins (V)	1.31 (1.06, 1.63)	0.01	1.45 (1.00, 2.15)	0.05	0.99 (0.48, 2.07)	0.99
90° to veins (V90)	1.53 (0.68, 3.14)	0.26	1.02 (0.35, 3.12)	0.92	2.22 (0.88, 4.92)	0.06
Surface‐in gradients and WM tract (GW)	0.63 (0.21, 1.86)	0.40	0.66 (0.05, 8.50)	0.75	0.93 (0.33, 2.63)	0.89
Surface‐in gradients, WM tracts and veins (GWV)	1.08 (0.78, 1.49)	0.64	1.01 (0.54, 1.89)	0.98	0.98 (0.67, 1.44)	0.93
Surface‐in gradients, WM tracts and 90° to veins (GWV90)	NA	NA	NA	NA	1.32 (0.07, 7.15)	0.79
None	1.42 (0.99, 2.02)	0.05	1.38 (0.70, 2.70)	0.93	1.20 (0.79, 1.83)	0.39

CI, confidence interval; MTR, magnetization transfer ratio; NA, no possible estimation due to the low number of lesions in each group; SD, standard deviation.

### Direction of expanding and contracting lesions

Table 4 summarizes the mean percentage of lesions per patient expanding or contracting along the four main orientations. Compared with chance alone, lesions expanding exclusively in alignment with one feature did so more frequently along WM tracts (W), veins (V) and orthogonally to veins (V90), while they contracted more frequently orthogonal to veins and less frequently along surface‐in gradients. Please see Figure S4 and Table S4 comparing the proportions of lesions expanding and contracting along the different orientations based on a 30° rather than a 45° threshold. Overall, the 30° threshold increased the proportion of lesions with no dominant direction of expansion and contraction. It did not materially change the relative proportion of lesions expanding or contracting along WM tracts, surface‐in gradients or veins, while we observed a lower relative proportion of lesions contracting orthogonally to veins. No associations were found between the patient's percentage of expanding and contracting lesions, whether considered a whole or grouped by their direction of expansion or contraction, and changes in EDSS over time.

**Table 4:** Percentages of lesions per patient expanding or contracting in alignment with a given feature.

	Surface‐in gradients (G)	Parallel to WM tracts (W)	Parallel to veins (V)	Orthogonal to veins (V90)
Exclusive expansion (*p* values vs. expected by chance alone)	0% (NA)	18% (more) (<0.001)	20% (more) (0.03)	22% (more) (0.02)
Overall expansion (*p* values vs. expected by chance alone)	1% (less) (<0.001)	31% (0.64)	25% (0.37)	27% (0.69)
Shared alignment with other directions				
Surface‐in gradient (G)	–	1% (less) (0.04)	0% (less) (0.02)	1% (less) (0.01)
WM tracts (W)	–	–	8% (more) (0.02)	4% (more) (0.02)
Surface‐in gradient and WM tracts (GW)	–	–	0% (0.37)	1% (1.00)
Exclusive contraction (*p* values vs. expected by chance alone)	1% (less) (<0.001)	8% (0.76)	15% (1.00)	23% (more) (0.03)
Overall contraction (*p* values vs. expected by chance alone)	4% (less) (<0.001)	37% (more) (0.01)	40% (more) (0.02)	26% (less) (0.01)
Shared alignment with other directions				
Surface‐in gradient (G)	–	0% (less) (0.05)	3% (0.35)	0% (less) (0.03)
WM tracts (W)	–	–	25% (more) (<0.001)	5% (more) (0.05)
Surface‐in gradient and WM tracts (GW)	–	–	0% (0.65)	0% (0.65)

Overall expansion and contraction include all lesions aligned with a given feature, even if they also align with other features. Exclusive expansion and contraction only include lesions aligned with a given feature alone.

NA, not possible chi‐squared calculation due to zero values.

### Effect of volume, shape and orientation on MTR changes

Table 5 summarizes the associations between baseline lesion features and MTR changes over the 6‐month follow‐up. Higher lesion volume and orientation along WM tracts (W) were associated with decrease in MTR values within lesions over the 6 months of follow‐up.

**Table 5:** Baseline predictors of lesion MTR changes.

	Beta coefficients (95% CI)	*p* values
Lesion volume	−0.14 (−0.24, −0.06)	0.01
Lesion anisotropy	0.02 (−0.48, 0.53)	0.92
Lesion orientation		
Surface‐in gradients (G)	0.14 (−0.53, 0.81)	0.68
Surface‐in gradients and veins (GV)	0.24 (−0.03, 0.50)	0.08
Surface‐in gradients and 90° to veins (GV90)	0.42 (−0.38, 1.22)	0.30
WM tracts (W)	−0.56 (−0.96, −0.15)	0.007
WM tracts and veins (WV)	0.02 (−0.17, 0.20)	0.86
WM tracts and 90° to veins (WV90)	−0.26 (−0.77, 0.25)	0.33
Veins (V)	0.04 (−0.13, 0.22)	0.64
90° to veins (V90)	−0.34 (−0.75, 0.06)	0.09
Surface‐in gradients and WM tract (GW)	0.36 (−0.39, 1.11)	0.35
Surface‐in gradients, WM tracts and veins (GWV)	−0.07 (−0.35, 0.22)	0.63
Surface‐in gradients, WM tracts and 90° to veins (GWV90)	0.49 (−0.37, 1.36)	0.27
None	−0.22 (−0.57, 0.11)	0.18

CI, confidence interval; MTR, magnetization transfer ratio; WM, white matter.

### Treatment effect

**Figure 2:**
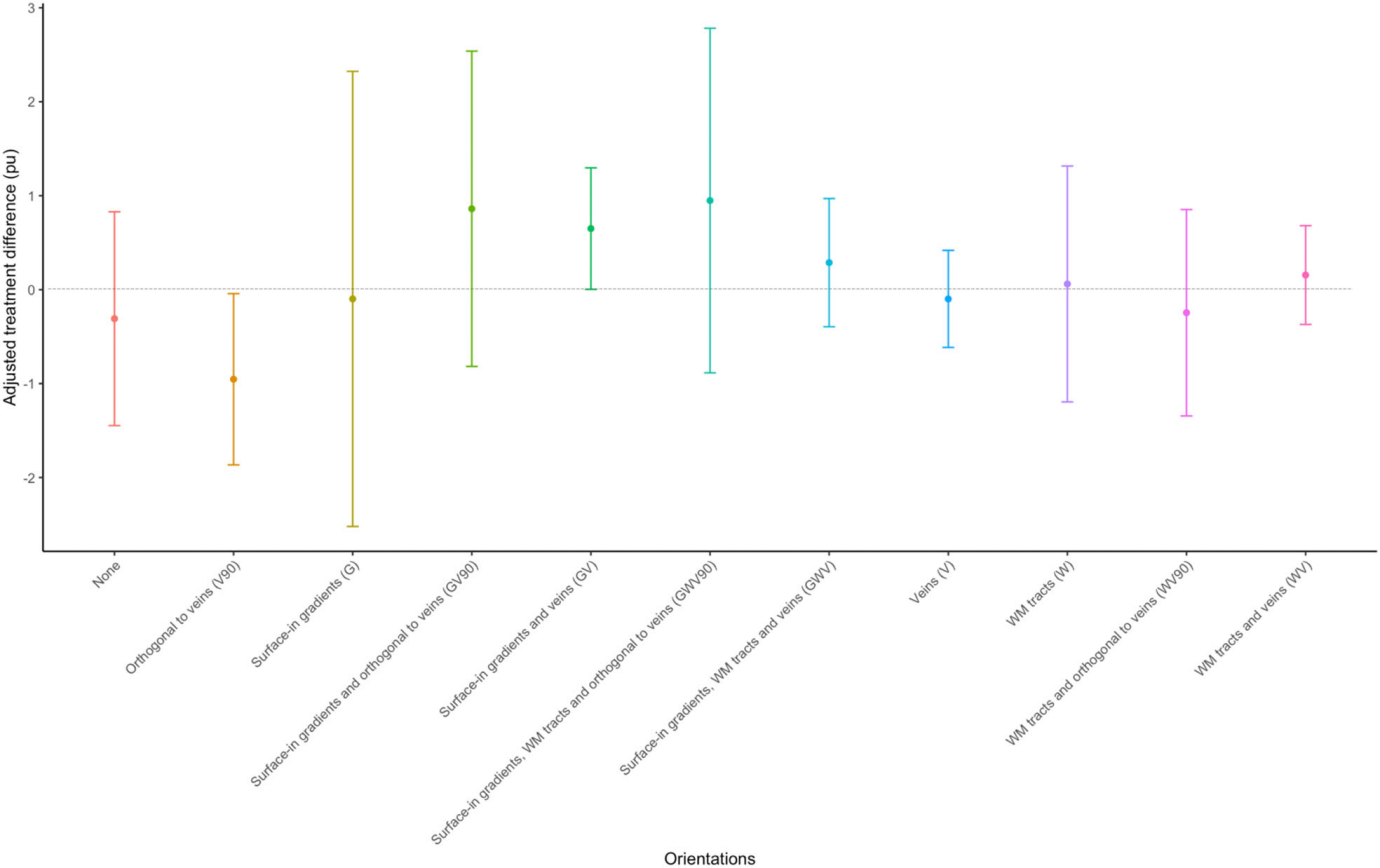
Adjusted bexarotene–placebo treatment differences in lesional MTR change, subdivided by lesion orientation. The plot summarizes the mean adjusted bexarotene–placebo treatment differences in lesional MTR change, subdivided by lesion orientation. The error bars represent 95% confidence intervals. MTR, magnetisation transfer ratio; pu, percentage units.

While treatment with bexarotene did not affect lesion expansion or contraction over the 6 months, in lesions orientated along surface‐in gradients and veins (GV) a positive treatment effect was seen, whereas for all the other lesion groups, it was not. Table 6 and Figure 2 summarize MTR changes in lesions grouped by bexarotene versus placebo according to their orientation.

**Table 6:** MTR changes in the placebo and bexarotene groups by baseline lesion orientation.

Lesion orientation	MTR change (Placebo group)	MTR change (Bexarotene group)	Bexarotene vs. Placebo	
	Unadjusted mean change (SD)	Unadjusted mean change (SD)	Adjusted difference (95% CI)	*p* values
Surface‐in gradients (G)	*N* = 2	*N* = 5		
	−0.12 (1.10)	0.10 (1.60)	0.14 (−1.71, 1.99)	0.99
Surface‐in gradients and veins (GV)	*N* = 55	*N* = 90		
	−0.10 (1.68)	0.30 (2.06)	0.63 (0.07, 1.20)	0.04
Surface‐in gradients and 90° to veins (GV90)	*N* = 5	*N* = 8		
	−0.05 (1.24)	0.78 (0.55)	0.86 (−0.82, 2.54)	0.31
WM tracts (W)	*N* = 14	*N* = 10		
	−0.63 (1.41)	−0.51 (1.21)	−0.20 (−1.12, 0.72)	0.67
WM tracts and veins (WV)	*N* = 146	*N* = 250		
	−0.05 (1.27)	0.08 (1.86)	0.19 (−0.38, 0.76)	0.51
WM tracts and 90° to veins (WV90)	*N* = 18	*N* = 14		
	−0.23 (0.94)	−0.40 (1.21)	0.95 (−0.89, 2.78)	0.66
Veins (V)	*N* = 169	*N* = 277		
	0.08 (1.51)	−0.10 (1.71)	−0.18 (−0.73, 0.38)	0.52
90° to veins (V90)	*N* = 21	*N* = 31		
	−0.22 (1.31)	−1.76 (2.13)	−0.85 (−1.87, 0.04)	0.08
Surface‐in gradients and WM tract (GW)	*N* = 1	*N* = 4		
	0.00 (1.47)	0.53 (1.72)	0.51 (−1.27, 2.30)	0.57
Surface‐in gradients, WM tracts and veins (GWV)	*N* = 42	*N* = 89		
	−0.29 (1.49)	−0.02 (1.92)	0.35 (−0.39, 1.09)	0.35
Surface‐in gradients, WM tracts and 90° to veins (GV90)	*N* = 4	*N* = 7		
	0.00 (1.47)	0.94 (2.13)	0.95 (−0.89, 2.78)	0.31
None	*N* = 14	*N* = 16		
	−0.09 (1.40)	−0.49 (2.94)	−0.21 (−1.03, 0.60)	0.60

CI, confidence interval; MTR, magnetization transfer ratio; *N*, number of lesions; SD, standard deviation.

### Lesions aligned with multiple features

Results are reported in Supplementary Materials (Tables S5–S7 and Figs. S2, S5 and S6).

## Discussion

In people with RRMS, we found that lesion orientation at baseline and subsequent lesion expansion and contraction show non‐random directionality. Using a novel lesionwise analysis, we show in vivo that lesion spatial dynamics have directional preferences that can be plausibly linked with pathogenic factors and that this influences the potential for tissue repair. While confirming lesion venocentric genesis (87% of lesions at baseline aligned with veins, ~33% exclusively so), we found that lesion expansion occurred along WM tracts, parallel and orthogonal to veins (all by ~5% more than expected by chance) suggesting that multiple factors influence this process. Treatment with bexarotene had no detectable effect on lesion expansion or contraction in any of the directions assessed, but an increase in MTR was observed in lesions orientated along both surface‐in and veins.

Before considering potential pathogenic interpretations of the findings, it is worth noting that we have presented orientation percentages for lesions in two ways: first, excluding lesions that are aligned with more than one feature, and second, including all lesions aligned with a given feature, even if they also align with another one (see Supplementary Materials). We base our interpretation of the results on the lesions exclusively associated with a given feature, as their occurrence beyond chance cannot be attributed to co‐linearity with other features. However, this is a conservative approach, and for lesions that align with more than one feature, it is possible that any combination of them may account for their orientation, and so we present the total percentage of lesions orientated with a given feature for context in Supplementary Materials. In addition, while it is reasonable to assume that lesion expansion represents more extensive demyelination, lesion contraction could potentially occur through remyelination or tissue collapse.^26^

It was unsurprising that most lesions showing directionality at baseline did so parallel to veins, and this is consistent with current models of T‐cell entry into brain parenchyma via veins being a key step in lesion genesis.^27^, ^28^, ^29^, ^30^ In contrast, while lesion expansion also occurred parallel to veins, it also did so orthogonal to them. It has been suggested that demyelination spreads radially from the central vein until the balance between pathogenic and repair factors reaches equilibrium, which could account for growth orthogonal to veins.^31^ A central role in limiting lesion growth appears to be played by perivenular fibrillar type I collagen deposition^14^ inhibiting CCL2‐dependent monocyte infiltration.^32^ It also appears to affect the morphology and the differentiation of both glial‐restricted progenitors and pre‐myelinating oligodendrocytes.^14^, ^33^ Interestingly, lesion contraction parallel to veins was not greater than chance, but orthogonal to veins it was. We specifically assessed growth orthogonal to veins in light of evidence suggesting that perivenular collagenosis may inhibit tissue repair, as vessel fibrosis can limit oligodendrocyte precursor cells' ability to reach the centre of demyelinated lesions and differentiate into mature oligodendrocytes.^14^, ^34^ Fibrillar collagen deposition may affect lesions in different ways over time, with collagen deposition contributing to eccentric thickening of the perivascular space early (it has been observed in demyelinating lesions less than 2 weeks old) and in the longer term vessel fibrosis (lesions older than 6 weeks) becomes more evident, with a limited proportion of mature oligodendrocytes reaching the lesion centre.^14^ The present results suggest that either collagenosis is not an inhibitor of remyelination or that lesion contraction observed in the present study is due to tissue collapse within the lesion rather than due to remyelination.^26^

At baseline, a substantial proportion of lesions were orientated along WM tracts, although when assessing lesions exclusively aligned with WM tracts, this was less than by chance alone, raising the possibility that collinearity with other features explained this. In contrast, lesion expansion along WM tracts was greater than by chance alone, suggesting that axonal degeneration may represent a relevant component of lesion expansion.^11^ A recent pathology study based on biopsies of people with MS with a disease duration of 1.9 years,^35^ found a significant association between neuropeptide Y receptor Y1 (NPY‐Y1R)‐positive axons within lesions and in the surrounding tissue, where anti‐NPY‐Y1R antibody is considered a label for Wallerian degeneration.^36^ In the same study,^35^ the number of NPY‐Y1R‐positive axons undergoing Wallerian degeneration was significantly higher in WM surrounding lesions than in extra‐lesional WM elsewhere. Furthermore, the immunopathological evidence of immunoglobulin targeting myelin and activated complement^5^, ^29^ suggests that an antigen‐specific immune response can spread along WM tracts, enhancing axonal degeneration arising from lesions.^37^, ^38^ Lesion contraction did not appear to be above chance alone parallel to WM tracts, suggesting that once tract‐mediated changes have occurred, they are not clearly reversible.

While we have concentrated on lesions showing alignment with one feature alone, it is possible that several may influence lesion growth and intrinsic tissue damage. Consistent with this, we found that lesions orientated along both WM tracts and parallel to veins were larger and had lower MTR values than those orientated along veins (reaching statistical significance) and those orientated along WM tracts (although not reaching statistical significance). A synergistic effect has been suggested by histopathological findings too, with a subpopulation of macrophages residing at the perivenular level in the CNS,^39^ and macrophage‐rich focal lesions with ongoing myelin degradation found at sites where Wallerian degeneration is most abundant.^40^

Looking at the baseline features predicting lesion expansion at follow‐up, we found that lower volume and anisotropy, and orientation along veins, were associated with a higher probability of lesion expansion over time. This might be explained by considering lesions based on their age: with chronic activity, we would expect increasing lesion size mainly occurring along veins or WM tracts, and so accompanied by increasing anisotropy, until factors promoting and opposing expansion reach an equilibrium. Hence, baseline WM lesions with lower volumes and anisotropy, especially if orientated along veins, are likely to be younger and so have more potential to expand over time.

So far, we have mainly considered the dynamics of lesion shape, but we also included a measure of lesion microstructure, MTR. Looking at the baseline features predicting lesion MTR changes at follow‐up, we observed that a higher lesion volume and orientation along WM tracts were associated with a decrease in MTR over time, suggesting that these are both associated with ongoing demyelination or axonal loss (all participants in CCMR One were clinically stable and taking dimethyl fumarate, making new inflammatory activity a less likely explanation).

Bexarotene has previously shown evidence for a remyelinating effect, as evidenced by an increase in MTR, clearly in GM lesions but also more subtly in WM lesion voxels (but not whole lesions) based on distance from the surface of the brain.^25^ In the present work, we did not observe any effect of bexarotene on WM lesion expansion or contraction over the 6 months' of observation, suggesting that lesion contraction is not a sensitive marker of remyelination. However, we did find a statistically significant treatment effect in lesions aligned along both surface‐in gradients and veins. To the best of our knowledge, this is the first time that therapeutic remyelination has been identified in whole WM lesions using only two scans: previously, we found a treatment effect in WM lesions aligned with surface‐in gradients only when assessing voxel‐wise changes. However, we did not observe a treatment effect in lesions exclusively orientated along surface‐in gradients (although there were only seven in the whole cohort) or parallel to veins (446 lesions in the whole cohort), and while we interpret this as indicating that alignment with surface‐in gradients influences remyelination potential at a whole lesion level, this result should be regarded with caution and requires replication. Further, given the small size of bexarotene's effect on WM lesion MTR compared to GM lesion MTR, exploring volume and MTR change in GM lesions would be of interest: the absence of dedicated GM imaging and small number of GM lesions detected (106 GM compared with 1613 WM lesions) precluded exploring this here.

This study has several other limitations. We approximated each lesion to an ellipsoid, which is likely to be less optimal for confluent lesions compared with solitary ones. However, even in confluent lesions, directionality is likely to be driven by the dominant component of the lesion, thus affecting our results less than systematically excluding a type of lesion that may be more susceptible to dynamic changes. As this was an exploratory study, we used a pragmatic minimum lesion size threshold of 3 mm^3^, which is commonly applied in MS. We did not set an additional threshold based on lesion anisotropy, as the minimum anisotropy value was already 0.27 units. Applying another threshold would likely not change the proportion of lesions relative to the total that aligns with a given feature, and thus our interpretation of the results, although it may reduce the absolute number of lesions included. If this approach is to be refined for use in treatment trials, a systematic assessment of both the volume threshold and the potential benefit of an anisotropy threshold would be necessary to optimize sensitivity to directional lesion growth. In the absence of SWI sequences to assess venous direction, we used an atlas, and we were not able to determine a venous orientation for 20% of lesions; compared with those in which we could, these lesions were smaller, with higher MTR values, less anisotropic and more likely to show an increase in MTR over time. As such, in future work, it would be of interest to try to include these lesions, given their greater likelihood of showing an MTR increase, as this may give further insights into factors promoting lesion repair. However, given their relative number, their omission is unlikely to have materially altered our main observations. Similarly, we used a WM DTI template due to the absence of DTI images, which limited the precision of identifying the true course of WM tracts because of natural inter‐individual differences. This likely introduced noise rather than bias. Additionally, because we assigned a single direction to lesions based on the atlas‐defined dominant tract, there may be a bias against detecting alignment with WM tracts in lesions containing a significant proportion of crossing fibres. However, this does not account for our finding of lesion expansion along WM tracts. If this method is to be used as a treatment outcome measure, lesions with a high proportion of crossing fibres may need to be excluded to avoid obscuring lesion growth along WM tracts. While the use of atlases can be considered a limitation, it allowed us to analyze lesion orientation independently from individual anatomical variants in this pilot study.

Preserving lesion shape was key to this analysis, as this was used to assess orientation. When registering to an MNI template, non‐rigid registrations are most often used, as they enable a closer alignment between boundaries, for example, periventricular WM and cerebrospinal fluid interface, compared with rigid registrations. They are typically undertaken using lesion‐filled images to avoid lesions distorting registration at boundaries, which is particularly relevant with periventricular lesions. However, features not seen on either the source or template images will not be specifically aligned, and so may be distorted. When we compared lesions that had been transformed based on non‐rigidly and rigidly registered lesion‐filled images, on inspection, we found that the non‐rigid registrations disrupted the shape of lesions noticeably more than with rigid registrations, and so we elected to use a rigid registration, accepting that there would be some misalignment with the vein and WM tract atlases. This misalignment will have been random across participants, should not have systematically biased the apparent lesion orientation relative to veins or WM tracts and so does not explain our findings. However, this will have added noise to orientation measures and so reduced the strength of associations of lesion orientations relative to each of the features assessed. Future work could seek to optimize registration methods and, ideally, directly compare with veins and WM tracts identified using dedicated MRI sequences in each participant as this would avoid the need to register to a template at all. We only had MRI data from two timepoints, preventing comparison with methods designed to detect slowly expanding lesions, which require three timepoints.^41^ Lesions were contoured on 2D sequences, a common practice in this field, likely limiting the precision of the Jacobian determinant calculation. Jacobian values can be influenced by changes in lesion intensity, potentially affecting volume measurements, but this is unlikely to account for the non‐random directional expansion or contraction observed. In addition, we developed our image analysis methods to answer questions about whether the directionality of lesions at baseline and their expansion or shrinkage was random or showed directional preferences. Given this, we considered lesions whose dominant axis was <45° from a given direction and based our interpretation of the results on lesions that only aligned with one feature. This gives confidence that lesion orientation is truly non‐random, and in our identification of the major features influencing this, but we are likely to be underestimating true effects by adopting such a conservative approach, particularly given that lesions can be affected by more than one factor simultaneously (and we find evidence for this), and that this resulted in some small groups (in particular only seven lesions were exclusively aligned with surface‐in gradients). From a clinical perspective, the limited follow‐up period did not allow us to observe significant changes in clinical disability, thereby reducing our ability to identify any associations between lesion directionality and the direction of lesion expansion or contraction with disability accrual. Lastly, as this study was exploratory, we did not adjust p‐values for multiple comparisons. Significant results at *p* ≤ 0.001 remain robust, but other findings should be interpreted with caution.

In conclusion, we observed that MS lesions show non‐random directionality at baseline and in their growth and contraction, indicating that the multiple (mainly vein and WM‐tract related) factors significantly influence this. Importantly, lesion shrinkage was not simply a reversal of lesion expansion, suggesting that some elements underlying lesion growth have an irreversible effect (in particular along WM tracts), and others may be more tractable to treatments. Bexarotene, which has shown evidence of a remyelinating effect in the present cohort, had no discernible effect on WM lesion growth or contraction, but lesion orientation was associated with lesional MTR change, again suggesting that some elements of pathology underlying lesion orientation (in this case perhaps most likely related to surface‐in gradients) are more responsive to treatments than others. In future work, it would be of interest to assess lesion orientation, expansion and contraction from first lesion formation rather than in already established lesions, over more than two time points and for longer than 6 months, as this would help us to better understand the dynamics of lesion growth and when each factor has its greatest effect. We used an atlas‐based approach in this exploratory study, but having demonstrated that WM tract and veins plausibly explain elements of lesion orientation, a direct evaluation of lesion directionality relative to WM tracts and veins based on dedicated scans would be valuable, both to confirm the present findings and to determine if this improves sensitivity to directional lesion expansion and contraction, which would be relevant if this approach is being optimized as a treatment trial outcome measure. It would also be of particular interest to study this in a mixed cohort of people with RRMS and secondary progressive MS, as histopathological and MRI studies suggest that lesion activity is likely greater than that observed in the present RRMS cohort.

## Conflict of Interest

Ermelinda De Meo, Alasdair J. Coles, Nick G. Cunniffe, Amy E. Jolly, Baris Kanber and Rebecca Samson report no conflict of interest. Ferran Prados is funded by the NIHR Biomedical Research Centre at UCL and UCLH. J. William L. Brown reports personal fees from Biogen for real‐world evidence consultation, outside the submitted work. Frederik Barkhof is part of the steering committee or Data Safety Monitoring Board member for Biogen, Merck, ATRI/ACTC and Prothena. He is a consultant for Roche, Celltrion, Rewind Therapeutics, Merck, IXICO, Jansen and Combinostics. He has research agreements with Merck, Biogen, GE Healthcare and Roche and is co‐founder and shareholder of Queen Square Analytics Ltd; reports consultancy fees from Roche, IXICO and Combinostics; grants from Biogen (progressive multifocal leukoencephalopathy educational website) and personal fees from Merck (steering committee), Prothena (data and safety monitoring board membership), Biogen (steering committee) and Eisai (data and safety monitoring board membership) outside the submitted work. Declan Chard is a consultant for Hoffmann‐La Roche. In the last 3 years, he has been a consultant for Biogen, has received research funding from Hoffmann‐La Roche, the International Progressive MS Alliance, the MS Society, the Medical Research Council, the National Institute for Health Research (NIHR) University College London Hospitals (UCLH) Biomedical Research Centre, and a speaker's honorarium from Novartis. He co‐supervises a clinical fellowship at the National Hospital for Neurology and Neurosurgery, London, which is supported by Merck.

## Author Contributions

EDM and DC contributed to study design, data analysis and drafting/revising the manuscript; FP contributed to data analysis and drafting/revising the manuscript; JWLB, AJC and NCG contributed to data collection and drafting/revising the manuscript; AEJ, BK, RS and FB contributed to drafting/revising the manuscript.

## Supporting information


Data S1.

## Data Availability

Requests for access to the data can be made to the trial chief investigator (Alasdair J. Coles).
