# Supplementary material for: An MRI assessment of mechanisms underlying lesion growth and shrinkage in multiple sclerosis
Source: Ann Clin Transl Neurol. 2025 Jan 27;12(4):686–700. doi: 10.1002/acn3.52308 (PMC12040513; doi:10.1002/acn3.52308)
Supplement: Supplementary file 1 — Data S1. [file ACN3-12-686-s001.pdf]

Supplementary material

- **MRI acquisition parameters**
- **Lesion segmentation procedure**
- **Orientation by chance alone calculation**
- **Table 1**
- **Figure 1**
- **Table 2**
- **Lesion features according to the four main orientations**
- **Figure 2**
- **Lesion features according to all the orientations**
- **Table 3**
- **Figure 3**
- **Figure 4**
- **Table 4**
- **Table 5**
- **Figure 5**
- **Table 6**
- **Table 7**
- **Figure 6**

MRI acquisition parameters

A Siemens 3T Prismafit scanner (Siemens, Erlangen, Germany) was used at each site with 20-channel head–neck coils. Baseline and month 6 scans included the following sequences: 3D magnetization transfer imaging [for calculation of MTR maps: voxel size 1 × 1 × 1 mm, TR = 35 msec, TE = 4.07/9.49 msec, flip angle 9°], 3DT1 weighted (for volumetric measures and segmentation: 1 × 1 × 1 mm, TR = 2400 msec, TE = 2.99 msec, flip angle = 8°), interleaved proton-density/T2-weighted scans (for identification and contouring of T2 hyperintense lesions: 1 × 1 × 3 mm, TR = 3050 msec, TE = 31/82 msec), fluid-attenuated-inversion recovery (FLAIR, to assist with lesion identification: 1 × 1 × 3 mm, TR = 9500 msec, TE = 123 msec) and post-gadolinium T1-weighted spin echo (to identify active lesions, 1 × 1 × 3 mm, TR = 600 msec, TE = 8.4 msec).

Lesion segmentation procedure

Baseline hyperintense lesions were identified on T2 sequences with the help of FLAIR using JIM v 6.0 (Xinapse systems, Aldwincle, UK). They were identified by a blinded rater, contoured by a different blinded rater and checked by a further blinded rater. Baseline lesion masks were then overlaid on the 6-month follow-up scans to ensure that the same tissue was examined at both timepoints. Lesion location was determined by tissue type based on the segmentation of 3D T1-weighted images after lesion-filling using a patch-based method[1] into white matter (WM), deep grey matter (DGM) and cortical grey matter (CGM), using the geodesic information flows.[2] T2-weighted scans were registered to 3D T1-weighted images, and the corresponding T2-weighted lesion masks then overlaid to allow each lesion's location to be classified. Due to the small number of GM lesions identified (106 as reported in the original trial manuscript)[3], in the present study we analysed pure WM lesions (with 100% of voxels belonging to WM) only. We limited our analysis to lesions with volume ≥ 3mm^3.[4]

Orientation by chance alone calculation

To calculate the probability of orientation of each lesion along a specific direction by chance alone as first we considered the probability of each lesion to form a 45° angle with a specific direction.

The angle between two random 3D vectors corresponds to the spherical surface area at that angle. Assuming a uniform distribution of directions the probability density function of the angle θ is proportional to the surface area of the spherical cap that subtends that angle and is calculated as: $f(\theta) = \sin(\theta)$, with θ ranging form 0 and π.

To find the probability of an angle less than or equal to 45°, we integrated the probability density function from 0 to $\pi/4$ as follows:

$$P\left(\theta < \frac{\pi}{4}\right) = \int_0^{\frac{\pi}{4}} \sin(\theta)\, d(\theta) \approx 0.2929.$$

Once we calculated the probability for a lesion to form a < 45° angle with a specific direction we considered the overlap among the different orientation. For each white matter (WM) voxel we calculated WM tract, surface-in gradients, vein and orthogonal to vein eigenvectors (as detailed in the main text) and we calculated the angle between each pair of eigenvectors. Again, to consider the overlap between the different directions we used a 45° angle threshold. With this approach the overlap between surface-in gradient and WM direction was 31%, thus resulting in a probability of 9% for a lesion to be orientated along surface-in gradient and WM direction by chance alone. The overlap between surface-in gradient and venous direction was 26%, thus resulting in a probability of 8% for a lesion to be orientated along surface-in gradient and vein direction by chance alone. The overlap between surface-in gradient and WM tract direction was 46%, thus resulting in a probability of 13% for a lesion to be orientated along WM and vein direction by chance alone. The overlap between surface-

in, WM and venous direction was 13%, thus resulting in a probability of 3% for a lesion to be orientated along surface-in gradient, WM and vein direction by chance alone.

Table 1. Baseline lesion features in lesions for whom was not possible to estimate vein direction, compared to the remaining ones.

	**Lesions with possible estimation of vein direction**	**Lesions with not possible estimation of vein direction**	***p* values**
Mean volume (SD) [ml]	65.39 (28.24)	18.54 (13.74)	<0.001
Mean MTR (SD) [pu]	43.53 (78.76)	43.91 (13.74)	0.05
Mean anisotropy (SD)	0.67 (0.17)	0.65 (0.16)	0.03
Orientation* G/GW/W/N	168/137/447/529	36/42/154/100	<0.001
Expansion/Contraction /Stable	171/92/1018	46/47/239	<0.001
Mean MTR change (SD) [pu]	-0.04 (1.75)	0.14 (1.85)	0.03
Direction of expansion* G/GW/W/N	2/3/54/112	1/2/27/16	0.63
Direction of contraction* G/GW/W/N	5/0/27/60	1/1/8/37	0.14

*Excluding vein and orthogonal to vein directions.

Abbreviations: SD=Standard deviation; MTR=magnetization transfer ratio; G=surface-in gradients; GW=surface-in gradients and white matter tracts; W=white matter tracts; N=none.

Lesion features according to the four main orientations

Including shared alignments, compared to the remaining orientations, lesions orientated along veins showed higher volume [adjusted difference=28.71ml 95% confidence interval (CI)=14.17ml, 43.37ml; $p<0.001$] and anisotropy (adjusted difference=0.14; 95%CI=0.12, 0.17; $p<0.001$) and lower MTR (adjusted difference=-0.97pu; 95%CI=-1.46pu, -0.48pu; $p<0.001$); lesions orientated orthogonally to veins showed lower anisotropy (adjusted difference=-0.10; 95%CI=-0.14, -0.07; $p<0.001$); while lesions without a specific orientation showed lower volume (adjusted difference=-34.89ml; 95%CI=-68.81ml, -2.07ml; $p=0.04$) and anisotropy (adjusted difference=-0.20; 95%CI=-0.26, -0.14; $p<0.001$).

Figure 1. Lesion orientation according to 30° vs 45° angle threshold.

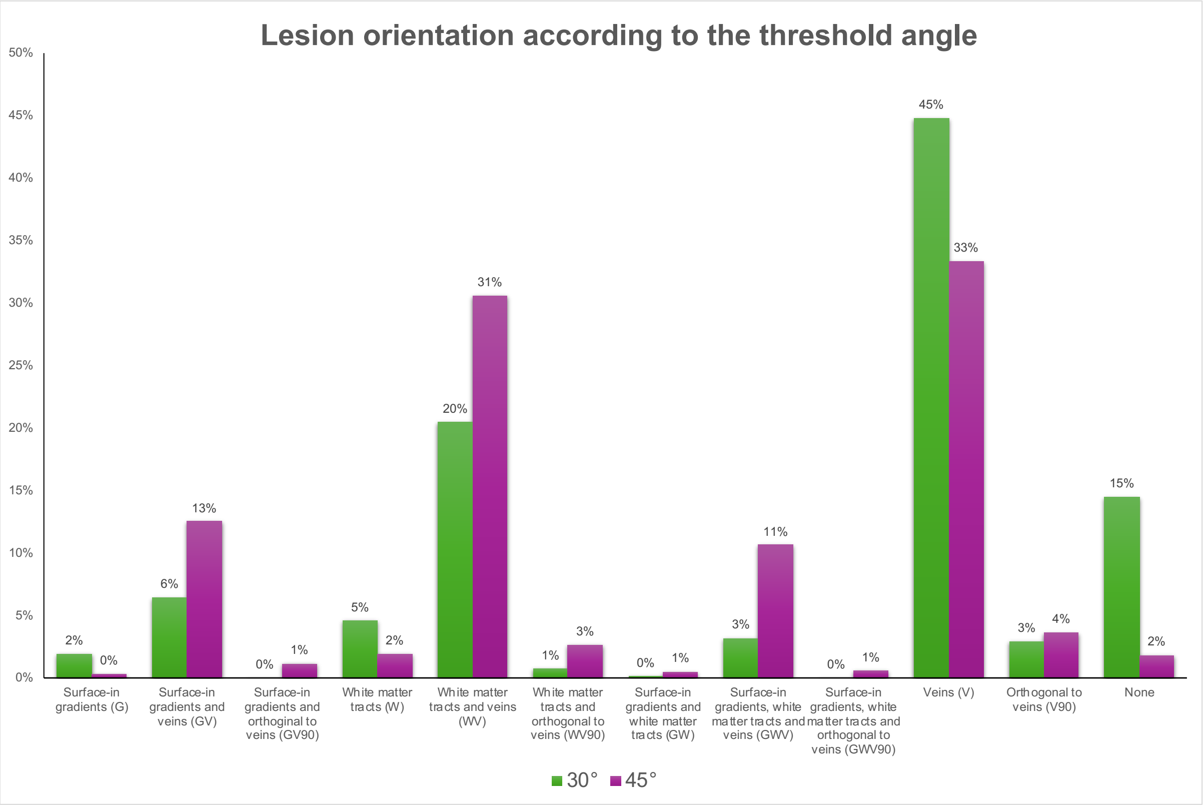

Table 2. Percentages of chance alone and observed lesion orientations at baseline according to 30° *vs* 45° angle threshold.

			30°				45°			
			**Surface-in gradients (G)**	**Parallel to WM tracts (W)**	**Parallel to veins (V)**	**Orthogonal to veins (V90)**	**Surface-in gradients (G)**	**Parallel to WM tracts (W)**	**Parallel to veins (V)**	**Orthogonal to veins (V90)**
**Expected exclusive chance orientation**			5%	5%	6%	6%	10%	10%	15%	15%
**Expected overall chance orientation**			7%	7%	7%	7%	29%	29%	29%	29%
	Shared alignment with other directions	Surface-in gradient (G)	-	1%	1%	1%	-	5%	6%	6%
		WM tracts (W)	-	-	1%	1%	-	-	4%	2%

		Surface-in gradient and WM tracts (GW)	-	-	0%	0%	-	-	1%	1%
**Observed exclusive lesion orientation (*p* values *vs* chance alone**			2% (less) (<0.001)	5% (1.00)	45% (more) (<0.001)	3% (less) (<0.001)	0% (less) (<0.001)	2% (less) (<0.001)	33% (more) (<0.001)	4% (less) (<0.001)
**Observed overall lesion orientation (*p* values *vs* chance alone)**			11% (more) (<0.001)	29% (more) (<0.001)	74% (more) (<0.001)	5% (more) (<0.001)	26% (less) (0.02)	46% (more) (<0.001)	87% (more) (<0.001)	8% (less) (<0.001)
	Shared alignment with other directions	Surface-in gradient (G)	-	0% (less) (<0.001)	6% (more) (<0.001)	0% (less) (<0.001)	-	1% (less) (<0.001)	13% (more) (<0.001)	1% (less) (<0.001)
		WM tracts (W)	-	-	20% (more) (<0.001)	1% (1.00)	-	-	31% (more) (<0.001)	3% (more) (0.01)
		Surface-in gradient and WM tracts (GW)	-	-	3% (more) (<0.001)	0% (1.00)	-	-	11% (more) (<0.001)	1% (0.99)

Abbreviations: WM=white matter.

Figure 2. Distribution of lesions according to their orientation

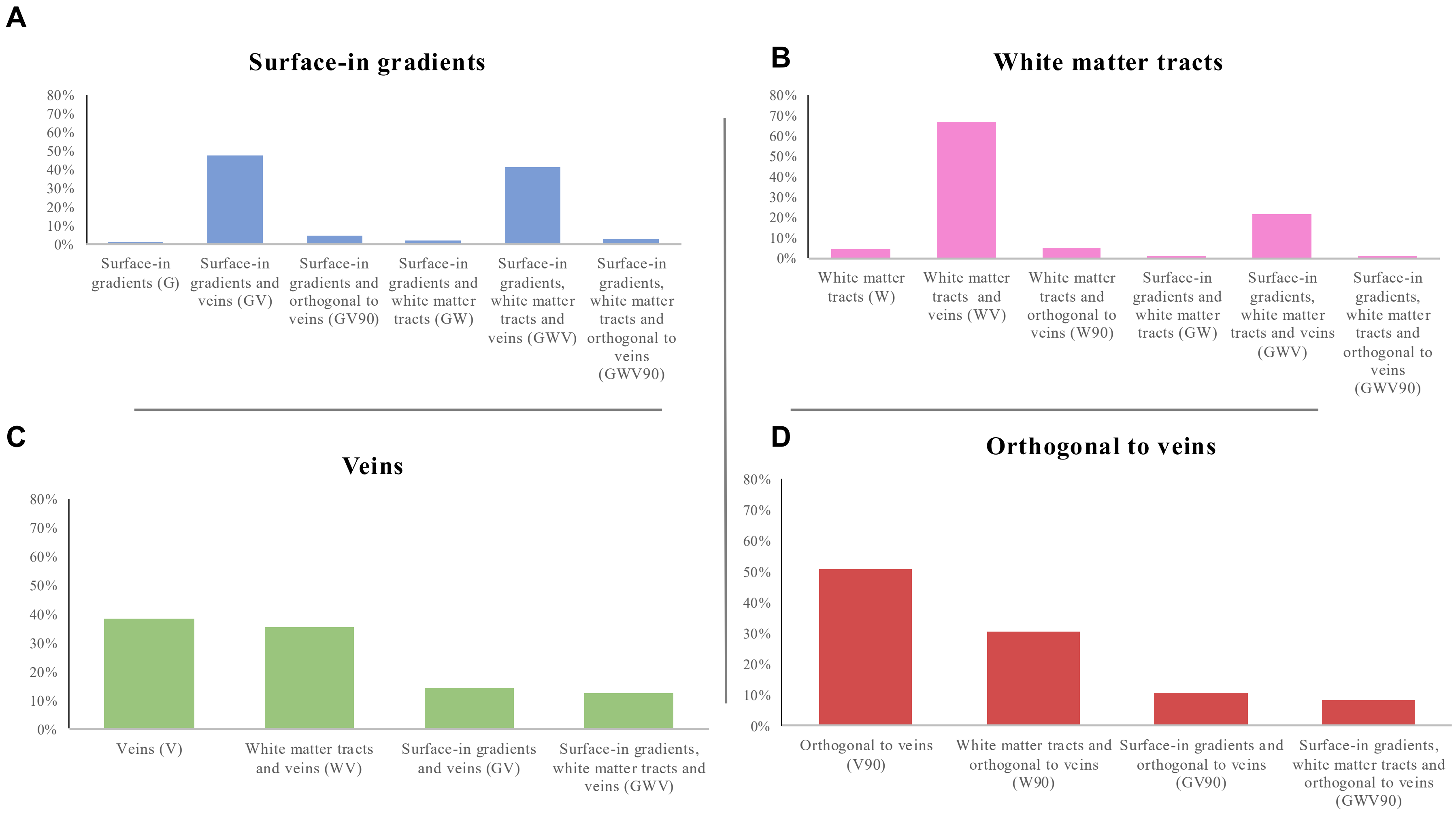

The barplots summarize the mean percentage of lesions per patient for each orientation grouped according the four main orientations **A**) Surface-in gradients, **B**) White matter tracts, **C**) Veins, **D**) orthogonal to veins

Lesion features according to all the orientations

Lesions orientated along WM tracts and veins (WV) had higher volume compared to all the remaining lesions together, to lesions without a specific orientation (N) and those orientated along all the remaining orientations except for surface-in gradient and WM tract (GW), surface-in gradient, WM tract and vein (GWV) and surface-in gradient, WM tract and orthogonal to venous direction (GWV90). Lesions orientated along surface-in gradients, WM tracts and veins (GWV) had higher volume compared to lesions without a specific orientation (N).

Lesions orientated along WM tracts and veins (WV) had lower MTR compared to all remaining lesions together and compared to lesions orientated along surface-in gradients (G), surface-in gradients and veins (GV), surface-in gradients and orthogonal to venous direction (GV90), WM tracts and orthogonal to venous direction (WV90), orthogonal to venous direction (V90), surface-in gradients and WM tracts (GW), and lesions without a specific orientation (N).

Lesions orientated along veins (V) showed higher anisotropy compared to all the remaining orientations except for surface-in gradient, WM tract and orthogonal to venous direction (GWV90). Lesions orientated along WM tracts and veins (WV) showed higher anisotropy compared to lesions without a specific orientation (N) and lesions orientated along all the remaining directions except for lesions orientated along surface-in gradients, WM tracts and veins (GWV). Lesions orientated along surface-in gradients, WM tracts and veins (GWV), showed higher anisotropy compared to lesions without a specific orientation (N) and all the remaining orientations except for lesions orientated along WM tracts and veins (WV).

Table 3. Baseline lesion features in lesions grouped by their orientation.

		Adjusted difference (95%CI) *p* values											
**Lesion volume [ml]**	**Mean (SD)**	***vs* all remaining groups**	***vs* surface-in gradients and veins (GV)**	***vs* surface-in gradients and 90° to veins (GV90)**	***vs* WM tracts (W)**	***vs* WM tracts and veins (WV)**	***vs* WM tracts and 90° to veins (WV90)**	***vs* veins (V)**	***vs* 90° to veins (V90)**	***vs* surface-in gradients and WM tract (GW)**	***vs* surface-in gradients, WM tracts and veins (GWV)**	***vs* surface-in gradients, WM tracts and 90° to veins (GWV90)**	***vs* none (N) *p* values**
**N=7 Surface-in gradients (G)**	25.75 (20.27)	-35.00 (-69.50, -0.43) 0.05	-32.25 (-87.75, 23.24) 0.64	-2.04 (-85.09, 81.01) 0.96	-17.29 (-77.71, 43.15) 0.98	-56.26 (-109.63, -2.94) 0.03	-21.83 (-95.68, 52.02) 0.56	-22.90 (-76.13, 30.23) 0.90	-15.43 (86.69, 55.82) 0.67	-19.85 (-97.94, 58.06) 0.99	-44.55 (100.72, 11.57) 0.23	-27.57 (-113.22, 58.07) 0.53	-10.98 (-68.74, 46.73) 0.99
**N=145 Surface-in gradients and veins (GV)**	56.94 (95.31)	-2.85 (-16.30, 10.60) 0.68		31.74 (-19.70, 83.17) 0.22	14.97 (-21.39, 51.34) 0.92	-24.01 (-46.21, -1.54) 0.03	11.95 (-22.68, 46.58) 0.50	9.35 (-12.79, 31.39) 0.90	18.34 (-10.22, 46.90) 0.21	12.40 (-48.95, 73.58) 0.99	-12.30 (-40.81, 16.18) 0.90	6.21 (-49.20, 61.61) 0.83	21.28 (-10.48, 52.97) 0.46
**N=13 Surface-in gradients and 90° to veins (GV90)**	26.19 (24.69)	-36.08 (-86.52, 13.78) 0.15			-12.03 (-73.27, 49.21) 0.70	-60.74 (-110.76, -10.73) 0.02	-19.79 (-78.11, 38.54) 0.51	-23.08 (-73.04, 26.88) 0.36	-13.39 (-68.44, 41.66) 0.63	-2.52 (-95.60, 90.55) 0.96	-43.30 (-94.90, 8.29) 0.09	-25.53 (-98.17, 47.11) 0.49	-1.24 (-60.11, 57.62) 0.97
**N=24 WM tracts (W)**	43.79 (39.57)	-18.30 (-39.10, 2.51) 0.08				-38.98 (-72.02, -5.99) 0.008	-7.75 (-55.74, 40.24) 0.75	-5.61 (-38.43, 27.09) 0.99	-1.36 (45.21, 42.49) 0.95	-2.57 (-68.52, 63.20) 1.00	-27.26 (-65.69, 10.11) 0.34	-13.51 (-78.21, 51.22) 0.68	6.31 (-33.62, 46.16) 1.00

**N=396<br>WM tracts and veins (WV)**	81.06 (102.48)	30.90<br>(21.70, 40.00)<br><0.001						33.37<br>(17.18, 49.49)<br><0.001	47.35<br>(21.92, 73.41)<br><0.001	36.41<br>(-23.02, 95.72)<br>0.57	11.71<br>(-12.44, 35.87)<br>0.82	35.21<br>(-18.98, 89.41)<br>0.20	45.29<br>(17.34, 73.22)<br><0.001
**N=32<br>WM tracts and 90° to veins (WV90)**	45.78 (48.17)	16.90<br>(-15.30 49.10)<br>0.30							6.39<br>(-33.42, 46.02)<br>0.75	17.26<br>(-68.03, 102.55)<br>0.69	-23.52<br>(-58.49, 11.45,)<br>0.19	-5.75<br>(-67.54, 56.05)<br>0.86	18.55<br>(-26.71, 63.79)<br>0.42
**N=446<br>Veins (V)**	46.71 (50.91)	-18.10<br>(-27.10, -9.16)<br><0.001							-0.55<br>(-1.43, 0.32)<br>0.21	3.04<br>(-56.27, 62.30)<br>1.00	-21.65<br>(-45.50, 2.25)<br>0.11	-2.45<br>(-56.57, 51.67)<br>0.93	11.92<br>(-15.71, 39.60)<br>0.89
**N=52<br>90° to veins (V90)**	39.65 (81.85)	23.8<br>(-1.57 49.2)<br>0.07								-0.07<br>(-2.87, 2.74)<br>0.96	-28.91<br>(-58.93, 0.89)<br>0.06	-12.14<br>(-71.03, 46.76)<br>0.69	12.15<br>(-28.68, 52.98)<br>0.56
**N=5<br>Surface-in gradients and WM tract (GW)**	44.04 (32.10)	-15.50<br>(-54.10, 23.10)<br>0.43									-24.70<br>(-86.54, 37.27)<br>0.92	-23.01<br>(-118.51, 72.50)<br>0.64	8.88<br>(-54.63, 72.49)<br>0.99
**N=131<br>Surface-in gradients, WM tracts**	68.37 (67.50)	10.6<br>(-4.08, 25.20)<br>0.16										17.77<br>(-37.88, 73.43)<br>0.53	33.57<br>(0.54, 66.59)<br>0.04

**and veins (GWV)**													
**N=11 Surface-in gradients, WM tracts and 90° to veins (GWV90)**	50.02 (36.22)	-10.61<br>(-64.76, 44.11)<br>0.70											24.29<br>(-38.24, 86.82)<br>0.45
**N=30 None**	33.90 (65.07)	-25.40<br>(-42.60, -8.16)<br>0.004											-
**Lesion MTR [pu]**	**Mean (SD)**	**Adjusted difference (95%CI)**<br>***p* values**											
**N=7 Surface-in gradients (G)**	44.78 (2.76)	1.58<br>(0.25, 2.92)<br>0.02	1.32<br>(-0.85, 3.50)<br>0.59	0.74<br>(-3.53, 2.06)<br>0.60	1.10<br>(-1.27, 3.47)<br>0.86	21.14<br>(0.05, 4.23)<br>0.04	0.62<br>(-1.86, 3.11)<br>0.62	1.36<br>(-0.73, 3.44)<br>0.50	0.47<br>(1.93, 2.86)<br>0.70	1.23<br>(-1.83, 4.29)<br>0.92	1.83<br>(-0.37, 4.03)<br>0.18	0.80<br>(-2.07, 3.68)<br>0.58	0.23<br>(-2.26, 2.72)<br>0.86
**N=145 Surface-in gradients and veins (GV)**	43.55 (3.36)	0.28<br>(-0.25, 0.80)<br>0.30		-1.71<br>(-3.44, -0.03)<br>0.05	-0.23<br>(-1.66, 1.20)<br>1.00	0.89<br>(0.07, 1.70)<br>0.05	0.34<br>(-1.52, 0.83)<br>0.56	0.03<br>(-0.83, 0.90)<br>1.00	0.50<br>(1.46, 0.46)<br>0.31	-0.09<br>(-2.50, 2.31)<br>1.00	0.51<br>(-0.61, 1.63)<br>0.86	0.40<br>(-2.83, 3.63)<br>0.81	-0.74<br>(-1.95, 0.47)<br>0.23
**N=13 Surface-in gradients**	45.15 (1.82)	2.00<br>(0.32, 3.69)			1.34<br>(-0.73, 3.41)	2.56<br>(0.87, 4.25)	1.36<br>(-0.61, 3.33)	1.76<br>(0.07, 3.45)	1.20<br>(0.65, 3.06)	-1.14<br>(-4.28, 2.01)	-2.27<br>(-4.01, -0.53)	1.54<br>(-0.91, 3.99)	0.97<br>(-1.02, 2.95)

**and 90° to veins (GV90)**		0.02			0.20	0.003	0.18	0.04	0.20	0.48	0.01	0.22	0.34
**N=24<br>WM tracts (W)**	43.86 (3.24)	0.50<br>(-0.31, 1.31)<br>0.23				1.04<br>(-0.25, 2.33)<br>0.21	0.02<br>(-1.60, 1.64)<br>0.98	0.26<br>(-1.02, 1.55)<br>1.00	-0.14<br>(-1.62, 1.34)<br>0.85	0.13<br>(-2.44, 2.71)<br>1.00	0.73<br>(-0.73, 2.20)<br>0.79	0.92<br>(-0.41, 2.26)<br>0.17	-0.38<br>(-2.01, 1.26)<br>0.65
**N=396<br>WM tracts and veins (WV)**	43.11 (3.60)	-0.82<br>(-1.18, -0.46)<br><0.001					-1.20<br>(-2.30, -0.10)<br>0.03	-0.78<br>(-1.41, -0.15)<br>0.004	-1.36<br>(-2.24, -0.48)<br>0.002	-0.91<br>(-3.23, 1.41)<br>0.94	-0.31<br>(-1.26, 0.64)<br>0.98	-1.02<br>(-2.85, 0.80)<br>0.27	-1.60<br>(-2.74, -0.46)<br>0.01
**N=32<br>WM tracts and 90° to veins (WV90)**	44.08 (2.60)	0.64<br>(-0.44, 1.72)<br>0.24						-0.49<br>(-0.70, -1.49)<br>0.48	-0.16<br>(-1.50, 1.19)<br>0.82	0.22<br>(-2.67, 3.11)<br>0.88	-0.91<br>(-2.09, 0.27)<br>0.13	0.18<br>(-1.90, 2.26)<br>0.87	-0.39<br>(-1.92, 1.13)<br>0.61
**N=446<br>Veins (V)**	43.74 (3.42)	0.32<br>(-0.03, 0.67)<br>0.07							-0.55<br>(-1.43, 0.32)<br>0.21	-0.13<br>(-2.45, 2.20)<br>1.00	0.48<br>(-0.46, 1.41)<br>0.78	-0.22<br>(-2.04, 1.60)<br>0.81	-0.79<br>(-1.93, 0.34)<br>0.17
**N=52<br>90° to veins (V90)**	44.15 (2.80)	0.80<br>(-0.05, 1.65)<br>0.06								-0.07<br>(-2.87, 2.74)<br>0.96	-0.98<br>(-2.04, 0.08)<br>0.06	0.34<br>(-1.65, 2.32)<br>0.74	-0.24<br>(-1.62, 1.14)<br>0.73
**N=5<br>Surface-in gradients**	44.01 (2.49)	0.36<br>(-1.14, 1.86)<br>0.63									0.60<br>(-1.82, 3.03)<br>1.00	0.40<br>(-2.83, 3.63)<br>0.81	-0.17<br>(-3.07, 2.73)<br>0.91

**and WM tract (GW)**													
**N=131 Surface-in gradients, WM tracts and veins (GWV)**	43.55 (3.36)	-0.28 (-0.85, 0.29) 0.33										-0.73 (-2.60, 1.14) 0.45	-1.30 (-2.52, -0.09) 0.04
**N=11 Surface-in gradients, WM tracts and 90° to veins (GWV90)**	44.00 (2.74)	0.43 (-1.39, 2.25) 0.64											-0.57 (-2.68, 1.53) 0.59
**N=30 None**	44.07 (2.88)	0.76 (0.09, 1.43) 0.03											
**Lesion anisotropy**	**Mean (SD)**	**Adjusted difference (95%CI)** ***p*** **values**											
**N=7 Surface-in gradients (G)**	0.56 (0.12)	-0.10 (-0.18, -0.03) 0.005	-0.06 (-0.17, 0.05) 0.71	-0.06 (-0.21, 0.08) 0.38	-0.01 (-0.14, 0.11) 1.00	-0.16 (-0.26, -0.05) <0.001	-0.09 (-0.21, 0.04) 0.19	-0.11 (-0.22, -0.00) 0.04	-0.02 (-0.14, 0.10) 0.74	0.05 (-0.11, 0.21) 0.98	-0.18 (-0.29, -0.06) <0.001	-0.07 (-0.22, 0.07) 0.32	0.07 (-0.06, 0.20) 0.27
**N=145**	0.63 (0.16)	-0.05 (-0.07,		0.03 (-0.06,	0.05 (-0.03,	-0.09 (-0.14,	0.01 (-0.05,	-0.05 (-0.09,	0.07 (0.02,	0.11 (-0.01,	-0.12 (-0.17,	0.02 (-0.08,	0.17 (0.10,

**Surface-in gradients and veins (GV)**		-0.02) <0.001		0.12) 0.52	0.12) 0.51	-0.05) <0.001	0.07) 0.79	-0.00) 0.02	0.12) 0.003	0.24) 0.10	-0.06) <0.001	0.11) 0.70	0.23) <0.001
**N=13 Surface-in gradients and 90° to veins (GV90)**	0.60 (0.13)	-0.07 (-0.17, 0.01) 0.09			0.05 (-0.05, 0.16) 0.32	-0.13 (-0.21, -0.04) 0.004	-0.02 (-0.12, 0.08) 0.68	-0.09 (-0.17, 0.00) 0.05	0.04 (-0.05, 0.14) 0.36	0.27 (0.11, 0.43) <0.001	-0.14 (-0.23, -0.05) 0.001	-0.01 (-0.14, 0.11) 0.87	0.14 (0.04, 0.24) 0.008
**N=24 WM tracts (W)**	0.58 (0.20)	-0.10 (-0.14, -0.05) <0.001				-0.14 (-0.20, -0.07) <0.001	-0.07 (-0.16, 0.01) 0.08	-0.09 (-0.16, -0.03) <0.001	-0.01 (-0.08, 0.07) 0.81	0.07 (-0.07, 0.20) 0.81	-0.16 (-0.24, -0.09) <0.001	-0.06 (-0.17, 0.05) 0.26	0.08 (0.00, 0.17) 0.05
**N=396 WM tracts and veins (WV)**	0.72 (0.15)	0.07 (0.05, 0.09) <0.001					0.11 (0.05, 0.16) <0.001	0.05 (0.01, 0.08) <0.001	0.17 (0.13, 0.22) <0.001	0.21 (0.09, 0.33) <0.001	-0.02 (-0.07, 0.03) 0.87	0.12 (0.02, 0.21) 0.01	0.26 (0.21, 0.32) <0.001
**N=32 WM tracts and 90° to veins (WV90)**	0.61 (0.17)	-0.06 (-0.12, 0.00) 0.04						-0.06 (-0.12, -0.01) 0.02	0.07 (0.00, 0.13) 0.05	0.29 (0.15, 0.44) <0.001	-0.12 (-0.18, -0.06) <0.001	0.01 (-0.10, 0.12) 0.84	0.16 (0.08, 0.24) <0.001
**N=446 Veins (V)**	0.67 (0.16)	0.01 (-0.01, 0.02) 0.56							0.13 (0.09, 0.17) <0.001	0.16 (0.04, 0.28) <0.001	-0.07 (-0.12, -0.02) <0.001	0.08 (-0.02, 0.17) 0.11	0.22 (0.16, 0.28) <0.001
**N=52**	0.55 (0.16)	-0.13								0.23	-0.18	-0.05	0.09

**90° to veins (V90)**		(-0.17,<br>-0.08)<br><0.001								(0.09,<br>0.37)<br><0.001	(-0.23,<br>-0.14)<br><0.001	(-0.16,<br>0.05)<br>0.29	(0.02,<br>0.16)<br>0.01
**N=5**<br>**Surface-in gradients and WM tract (GW)**	0.52 (0.20)	-0.16<br>(-0.24,<br>-0.08)<br><0.001									-0.23<br>(-0.35,<br>-0.10)<br><0.001	-0.28<br>(-0.45,<br>-0.12)<br><0.001	-0.14<br>(-0.28,<br>0.01)<br>0.07
**N=131**<br>**Surface-in gradients, WM tracts and veins (GWV)**	0.74 (0.15)	0.08<br>(0.05,<br>0.11)<br><0.001										0.13<br>(0.03,<br>0.23)<br>0.01	0.28<br>(0.22,<br>0.34)<br><0.001
**N=11**<br>**Surface-in gradients, WM tracts and 90° to veins (GWV90)**	0.61 (0.17)	0.07<br>(-0.17,<br>0.03)<br>0.17											0.15<br>(0.04,<br>0.25)<br>0.01
**N=30**<br>**None**	0.46 (0.14)	-0.16<br>(-0.20,<br>-0.13)<br><0.001											

Abbreviations: MTR=magnetization transfer ratio, SD=standard deviations, CI=confidence interval

Figure 3. Lesion features according to their orientation

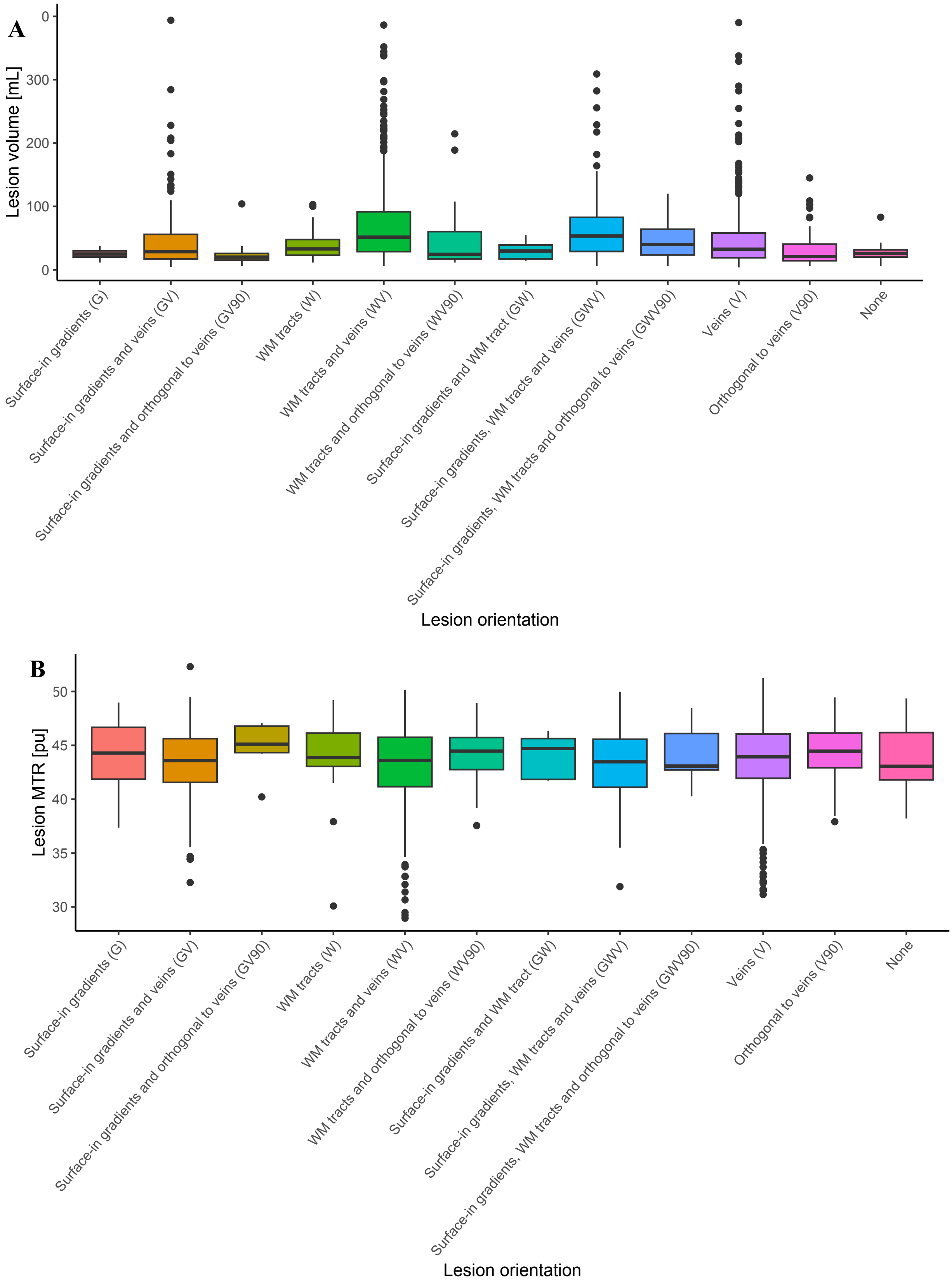

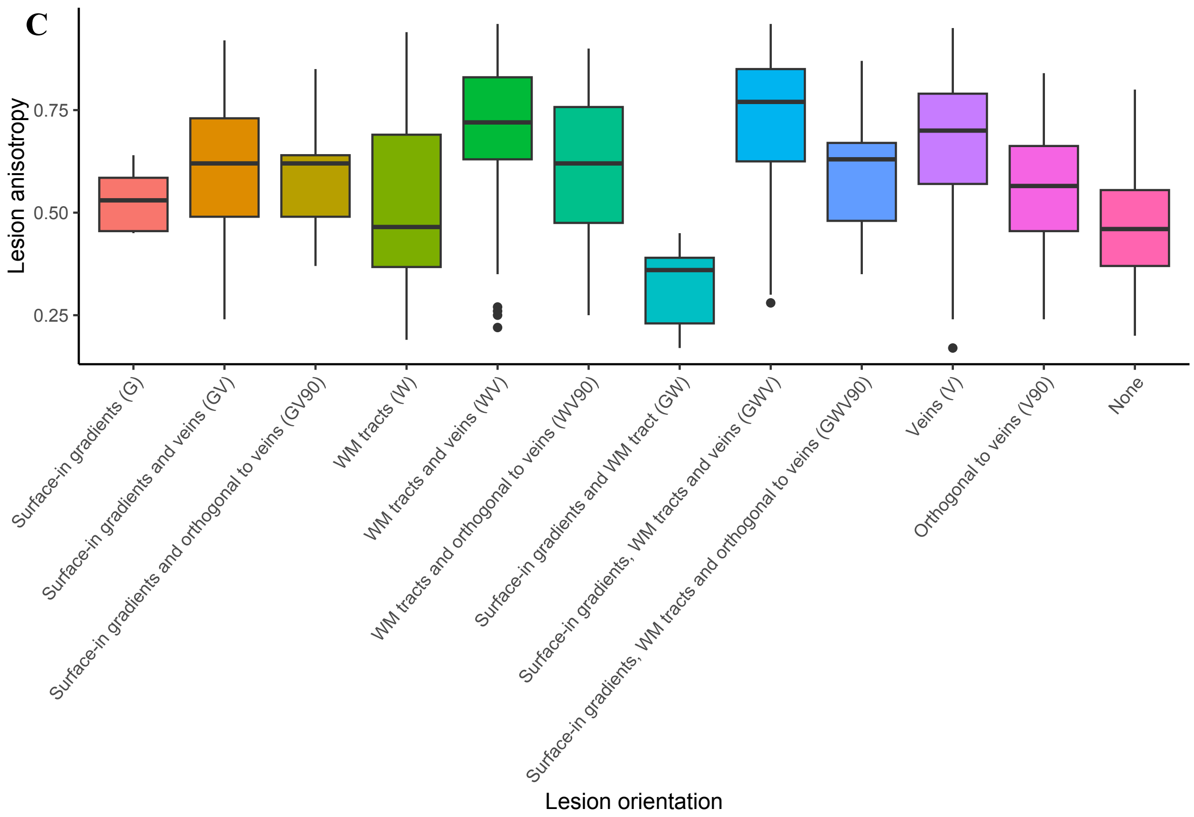

The boxplots summarize volume (A), magnetization transfer ratio (MTR) (B) and anisotropy (C) in lesions grouped according to their orientation.

Figure 4. Lesion direction of expansion and contraction according to 30° vs 45° angle threshold.

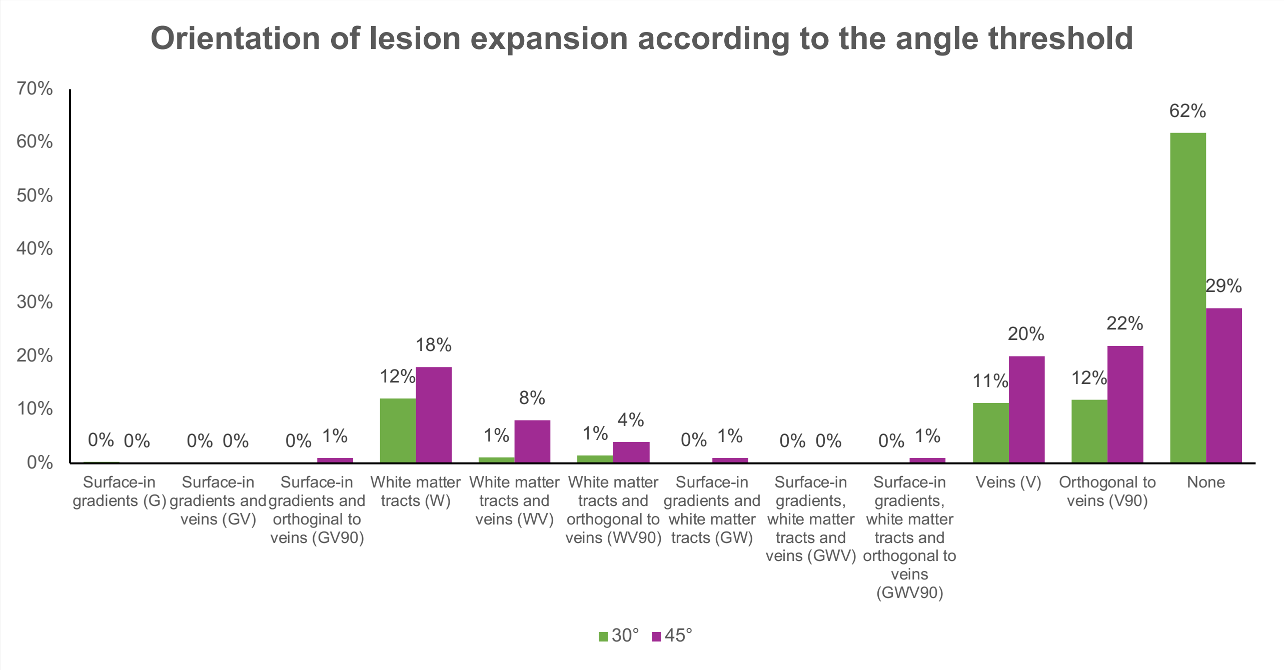

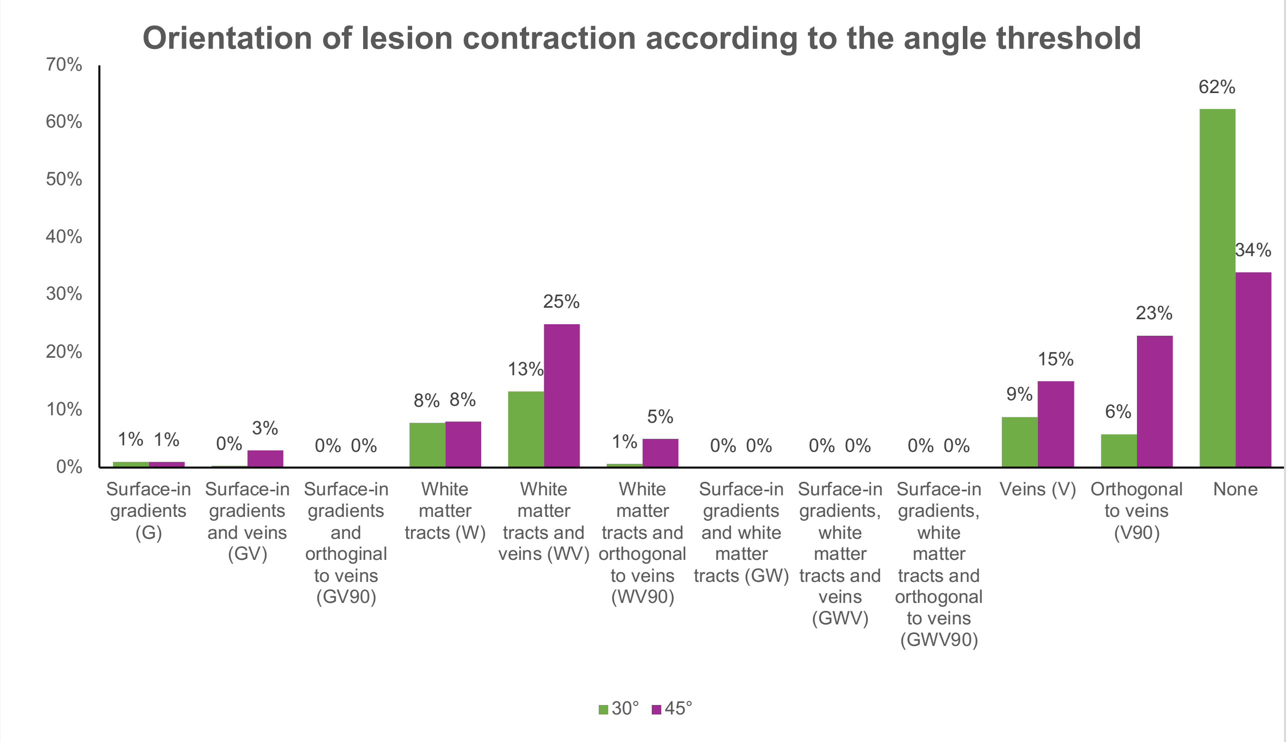

Table 4. Percentages of lesions per patient expanding or contracting in alignment with a given feature according to 30° *vs* 45° angle threshold.

		**30°**				**45°**			
		**Surface-in gradients (G)**	**Parallel to WM tracts (W)**	**Parallel to veins (V)**	**Orthogonal to veins (V90)**	**Surface-in gradients (G)**	**Parallel to WM tracts (W)**	**Parallel to veins (V)**	**Orthogonal to veins (V90)**
**Exclusive expansion (*p* values *vs* expected by chance alone)**		0% (less) (<0.001)	12% (more) (0.05)	11% (more) (0.04)	12% (more) (0.02)	0% (NA)	18% (more) (<0.001)	20% (more) (0.03)	22% (more) (0.02)
**Overall expansion (*p* values *vs* expected by chance alone)**		0% (less) (<0.001)	14% (0.16)	12% (0.07)	13% (more) (0.05)	1% (less) (<0.001)	31% (0.64)	25% (0.37)	27% (0.69)
Shared alignment with other directions	Surface-in gradient (G)	-	0% (less) (<0.001)	0% (less) (0.001)	0% (less) (<0.001)	-	1% (less) (0.04)	0% (less) (0.02)	1% (less) (0.01)
Shared alignment with other directions	WM tracts (W)	-	-	1% (1.00)	1% (1.00)	-	-	8% (more) (0.02)	4% (more) (0.02)

		Surface-in gradient and WM tracts (GW)	-	-	0% (1.00)	0% (1.00)	-	-	0% (0.37)	1% (1.00)
**Exclusive contraction (*p* values *vs* expected by chance alone)**			1% (less) (<0.001)	8% (0.32)	9% (0.43)	6% (1.00)	1% (less) (<0.001)	8% (0.76)	15% (1.00)	23% (more) (0.03)
**Overall contraction (*p* values *vs* expected by chance alone)**			1% 0.01	22% (more) (0.003)	22% (more) (0.003)	7% (1.00)	4% (less) (<0.001)	37% (more) (0.01)	40% (more) (0.02)	26% (less) (0.01)
	Shared alignment with other directions	Surface-in gradient (G)	-	0% (less) (<0.001)	0% (less) (0.001)	0% (less) (<0.001)	-	0% (less) (0.05)	3% (0.35)	0% (less) (0.03)
		WM tracts (W)		-	13% (more) (0.009)	1% (1.00)	-	-	25% (more) (<0.001)	5% (more) (0.05)
		Surface-in gradient and WM tracts (GW)	-	-	0% (1.00)	0% (1.00)	-	-	0% (0.65)	0% (0.65)

Abbreviations: WM=white matter.

Overall expansion and contraction include all lesions aligned with a given feature even if they also align with other features. Exclusive expansion and contraction only include lesions aligned with a given feature alone.

Table 5. Baseline main lesion orientation predicting lesion expansion or contraction over the 6-month follow-up.

**Lesion orientation**	Expanding *vs* Stable		Expanding *vs* Contracting		Contracting vs Stable	
	**Odds ratio (95% CI)**	***p* values**	**Odds ratio (95% CI)**	***p* values**	**Odds ratio (95% CI)**	***p* values**
Surface-in gradients	0.91 (0.60, 1.34)	0.63	0.77 (0.41, 1.46)	0.43	1.12 (0.68, 1.79)	0.65
WM tracts	0.60 (0.42, 0.84)	0.003	0.54 (0.30, 0.95)	0.04	0.83 (0.54, 1.27)	0.39
Veins	1.12 (0.69, 1.91)	0.66	0.83 (0.35, 1.88)	0.65	1.05 (0.58, 2.03)	0.88
Orthogonal to veins	0.75 (0.37, 1.40)	0.40	0.91 (0.34, 2.50)	0.86	1.17 (0.53, 2.29)	0.68
None	1.42 (0.99, 2.02)	0.05	1.38 (0.70, 2.70)	0.93	1.20 (0.79, 1.83)	0.39

Abbreviations: CI=confidence interval.

Figure 5. Direction of expansion and contraction

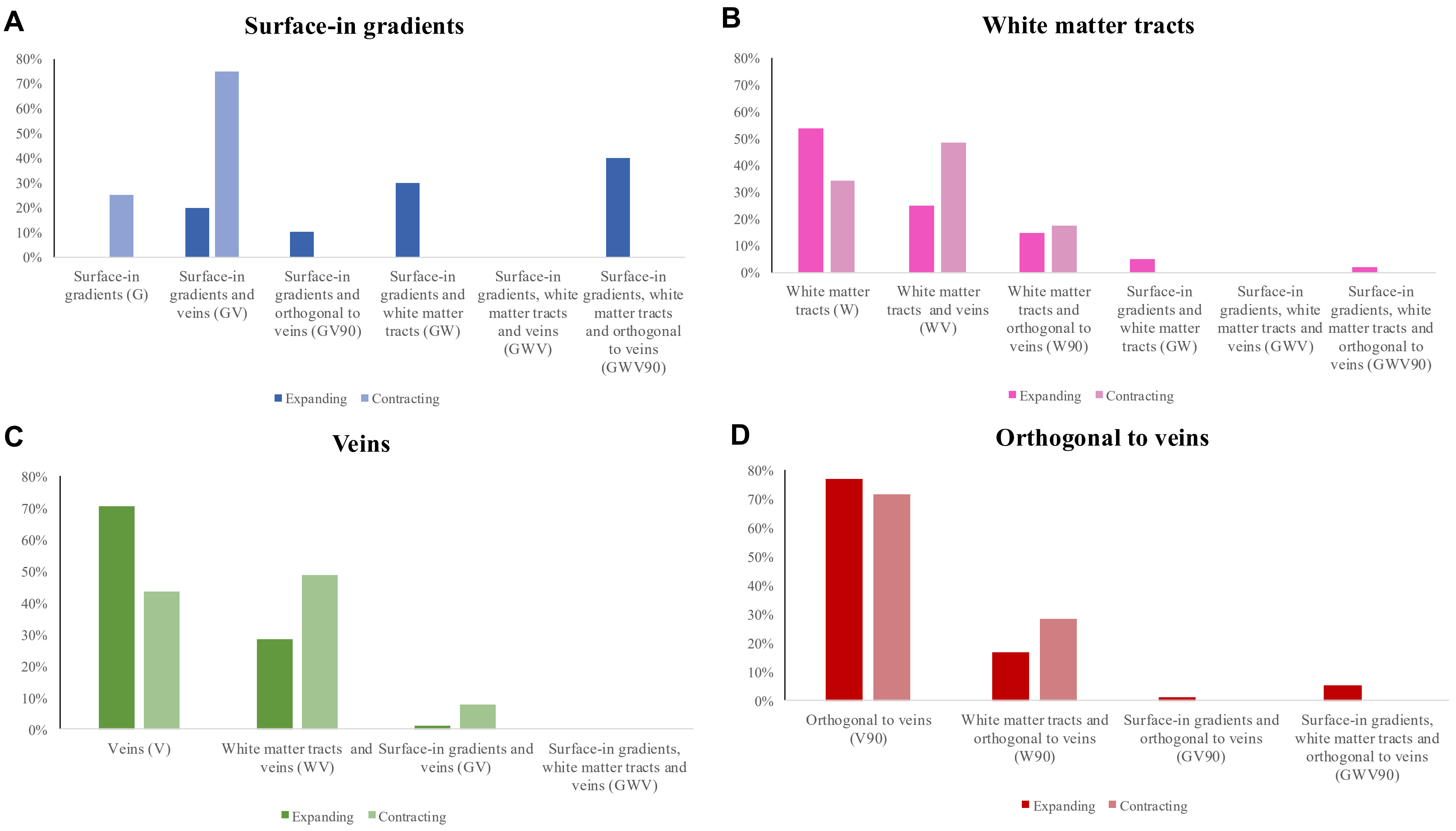

The barplots summarise the mean mutually exclusive percentage of lesions per patient that are expanding (shown in full colour) and contracting (shown in shaded colour) relative to each main orientation. The data is grouped according the four main orientations: **A**) Surface-in gradients, **B**) White matter tracts, **C**) Veins, **D**) orthogonal to veins.

Table 6. Summarizes main lesion orientation effect on MTR changes in the whole group of lesions.

**Lesion orientation**	**Beta coefficients (95% CI)**	***p* values**
Surface-in gradients	0.19 (0.01, 0.39)	0.04
WM tracts	-0.11 (-0.27, 0.05)	0.18
Veins (V)	0.37 (0.14, 0.61)	0.002
Orthogonal to veins (V90)	-0.37 (-0.65, -0.08)	0.01
None	-0.22 (-0.57, 0.11)	0.18

Abbreviations: MTR=magnetization transfer ratio, SD=standard deviations, CI=confidence interval.

Table 7. Summarizes the MTR changes in Placebo and Bexarotene group according to lesion orientation.

**Lesion orientation**	**MTR change (Placebo group)**	**MTR change (Bexarotene group)**	**Bexarotene *vs* Placebo**	
	Unadjusted mean change (SD)	Unadjusted mean change (SD)	Adjusted difference (95% CI)	*p* values
Surface-in gradients	N=201<br>-0.09<br>(1.56)	N=111<br>0.21<br>(1.94)	0.45<br>(0.01, 0.89)	0.01
WM tracts	N=375<br>-0.16<br>(1.34)	N=224<br>-0.05<br>(1.75)	0.21<br>(-0.32, 0.73)	0.43
Veins	N=706<br>-0.01<br>(1.42)	N=412<br>0.00<br>(1.78)	0.13<br>(-0.37, 0.62)	0.61
Orthogonal to veins	N=57<br>-0.18<br>(1.16)	N=51<br>-0.87<br>(2.10)	-0.41<br>(-1.12, 0.31)	0.26
None	N=16<br>-0.09<br>(1.40)	N=14<br>-0.49<br>(2.94)	-0.21<br>(-1.03, 0.60)	0.60

Abbreviations: MTR=magnetization transfer ratio, SD=standard deviations, CI=confidence interval.

Figure 6. **Adjusted bexarotene–placebo treatment differences in lesional MTR change, subdivided by lesion orientation.**

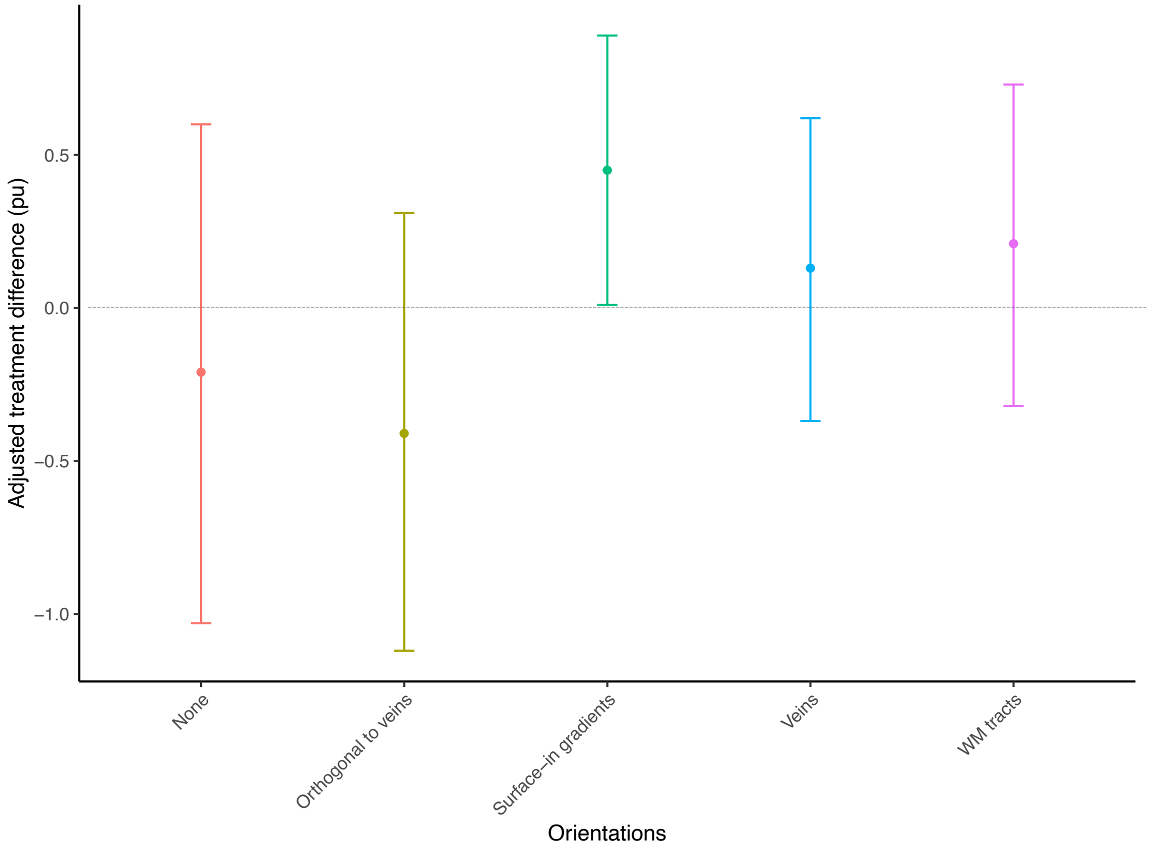

The plot summarizes the mean adjusted bexarotene–placebo treatment differences in lesional MTR change, subdivided by lesion orientation. The error bars represent 95% CIs. MTR=magnetisation transfer ratio. pu=percentage units.

References

1. Prados F, Cardoso MJ, Kanber B, et al. A multi-time-point modality-agnostic patch-based method for lesion filling in multiple sclerosis. Neuroimage 2016;139:376-384.

2. Cardoso MJ, Modat M, Wolz R, et al. Geodesic Information Flows: Spatially-Variant Graphs and Their Application to Segmentation and Fusion. IEEE Trans Med Imaging 2015;34:1976-1988.

3. Brown JWL, Cunniffe NG, Prados F, et al. Safety and efficacy of bexarotene in patients with relapsing-remitting multiple sclerosis (CCMR One): a randomised, double-blind, placebo-controlled, parallel-group, phase 2a study. The Lancet Neurology 2021;20:709-720.

4. Filippi M, Preziosa P, Banwell BL, et al. Assessment of lesions on magnetic resonance imaging in multiple sclerosis: practical guidelines. Brain 2019;142:1858-1875.
